# Supplementary material for: Timing the initiation of multiple myeloma
Source: Nat Commun. 2020 Apr 21;11:1917. doi: 10.1038/s41467-020-15740-9 (PMC7174344; doi:10.1038/s41467-020-15740-9)
Supplement: Supplementary file 1 — Supplementary Information [file 41467_2020_15740_MOESM1_ESM.pdf]

SUPPLEMENTARY INFORMATION

Timing the initiation of multiple myeloma

Rustad EH et al.

SUPPLEMENTARY METHODS

VDJ and HCDR3 reconstruction from WGS data

Reads overlapping V and J exons were extracted from bwa-mem mapped BAM files using coordinates from the wgEncodeGencodeBasicV19 table on GRCh37/hg19 genome build. We filtered out duplicate, supplementary, QC fail and secondary reads. To recover V-D-J junction sequences, we extracted soft clipped reads that overlapped exon boundaries. *IGH* is orientated in the reverse orientation relative to the genome build, and we extracted: (1) 5' clipped forward reads overlapping a V exon; (2) 3' clipped reverse reads overlapping a V exon; (3) 3' clipped forward reads overlapping a J exon; and (4) 5' clipped reverse reads overlapping a J exon. Candidate (V,J) exons were defined by correctly oriented paired-end reads, where one of the reads overlaps a V exon and its mate overlaps a J exon. For each candidate (V,J) combination, we built a directed graph from read kmers, where each node was a kmer and edges were added between adjacent kmers. Each kmer was also given a weight corresponding to the number of times it was observed. Initially, we built the directed graph with kmer length equals 11 but if the resulting graph had cycles, we increased the kmer length by two until the graph was acyclic. This yielded a directed acyclic graph (DAG). We then added an edge between a 'source' node and kmers from V exons with no incoming edges. We also added an edge between kmers from J exons with no outgoing edges and a 'sink' node. After building the DAG, we enumerated all possible paths between 'source' and 'sink' nodes. We reconstructed the nucleotide sequence from path kmers and translated it into all three reading frames,

before identifying the CDR3 as the region flanked by the final cysteine residue of the V gene and the conserved FGXG or WGXG motif in the J gene.

SUPPLEMENTARY FIGURES

Supplementary Figure 1. SBS-MM1 mutational signature extraction. The new mutational signature SBS-MM1 was extracted by SigProfiler (A/C) and *hdp* (B/D) from 96-classes (A-B) and 1536-classes mutational profiles, collapsed into 96-classes (C-D).

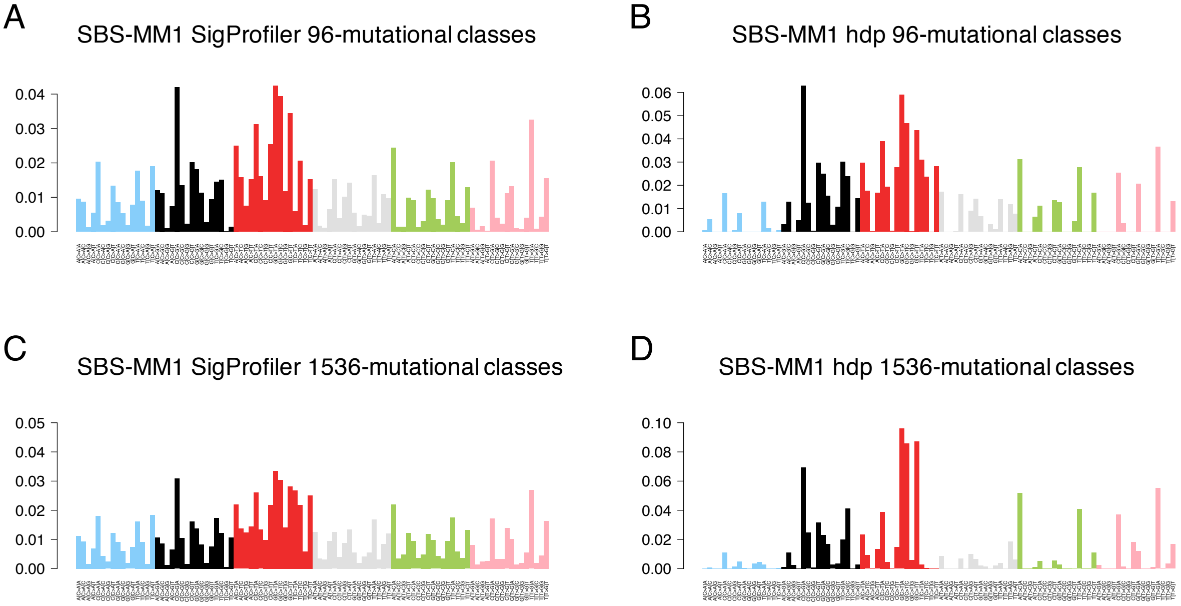

Supplementary Figure 2. SBS-MM1 contribution (dots) and 95% confidence intervals estimated by hdp-based *de novo* mutational signature extraction. All samples were run together, and here plotted separately according to the histology (see Methods). A significant contribution from SBS-MM1 was observed exclusively after melphalan exposure, in 9 patients with multiple myeloma and in the only human pluripotent cell line exposed to melphalan (MSM0.10) (A,B). In contrast, no significant SBS-MM1 contribution was observed in the absence of melphalan exposure, including patients with multiple myeloma who had not received melphalan (A), human pluripotent cell lines who were not exposed to chemotherapy (“Controls”; B), and patients with Chronic lymphocytic leukemia (CLL; C). CLL WGSs were included as negative control to estimate the inter-bleeding of signatures across different samples.

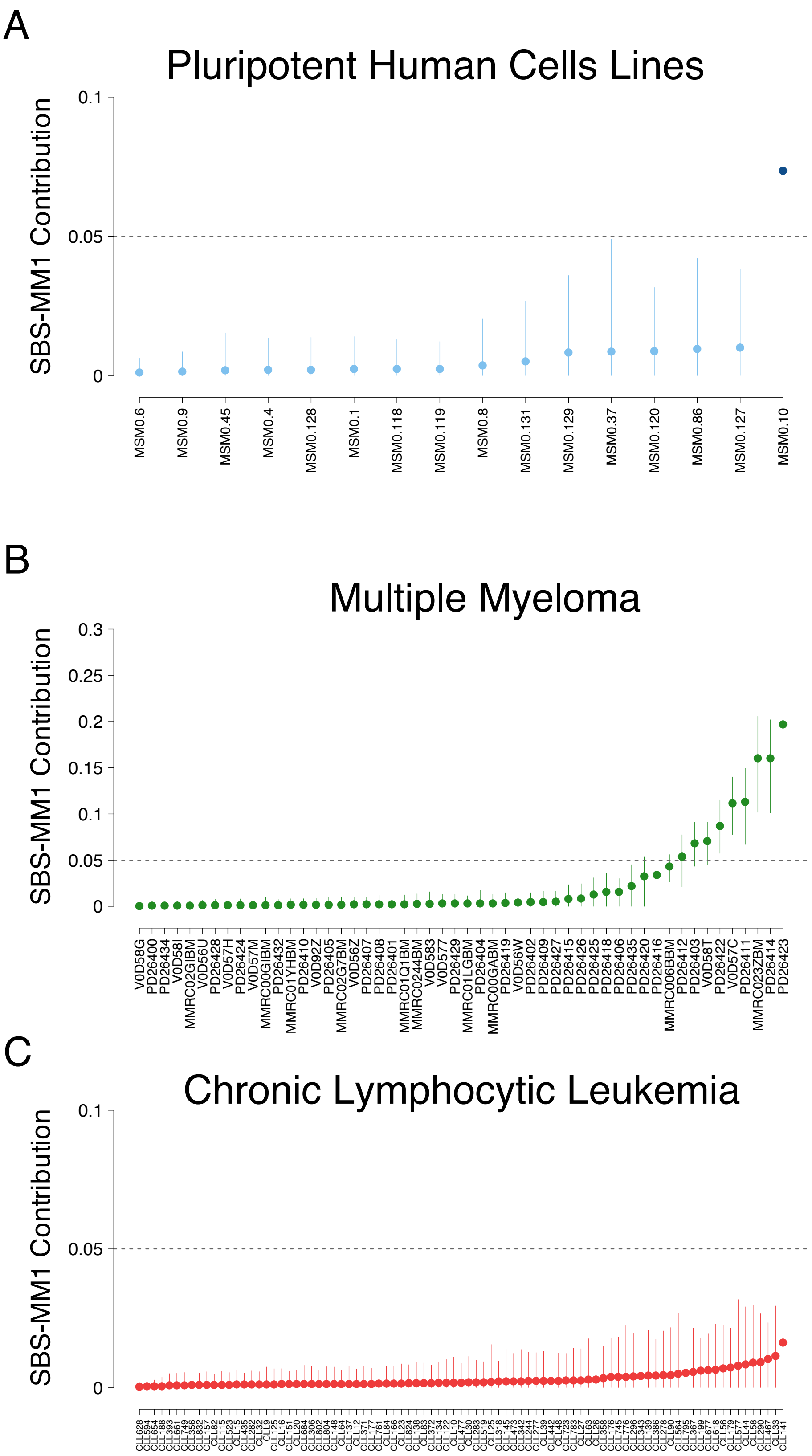
A
Pluripotent Human Cells Lines
SBS-MM1 Contribution
0.1
0.05
0
MSM0.6
MSM0.9
MSM0.45
MSM0.4
MSM0.128
MSM0.1
MSM0.118
MSM0.119
MSM0.8
MSM0.131
MSM0.129
MSM0.37
MSM0.120
MSM0.86
MSM0.127
MSM0.10
B
Multiple Myeloma
SBS-MM1 Contribution
0.3
0.25
0.2
0.15
0.1
0.05
0
C
Chronic Lymphocytic Leukemia
SBS-MM1 Contribution
0.1
0.05
0

Supplementary Figure 3. Mutational signature deconvolution of WGS data from human-induced pluripotent stem cells exposed to melphalan (MSM0.10)[2]. A) Decomposition of MM0.10 mutational profile according to the likelihood of each mutation to belong to SBS-MM1 or to the “Control” signature (i.e., the signature detected in all single cell expansions exposed to different agents).

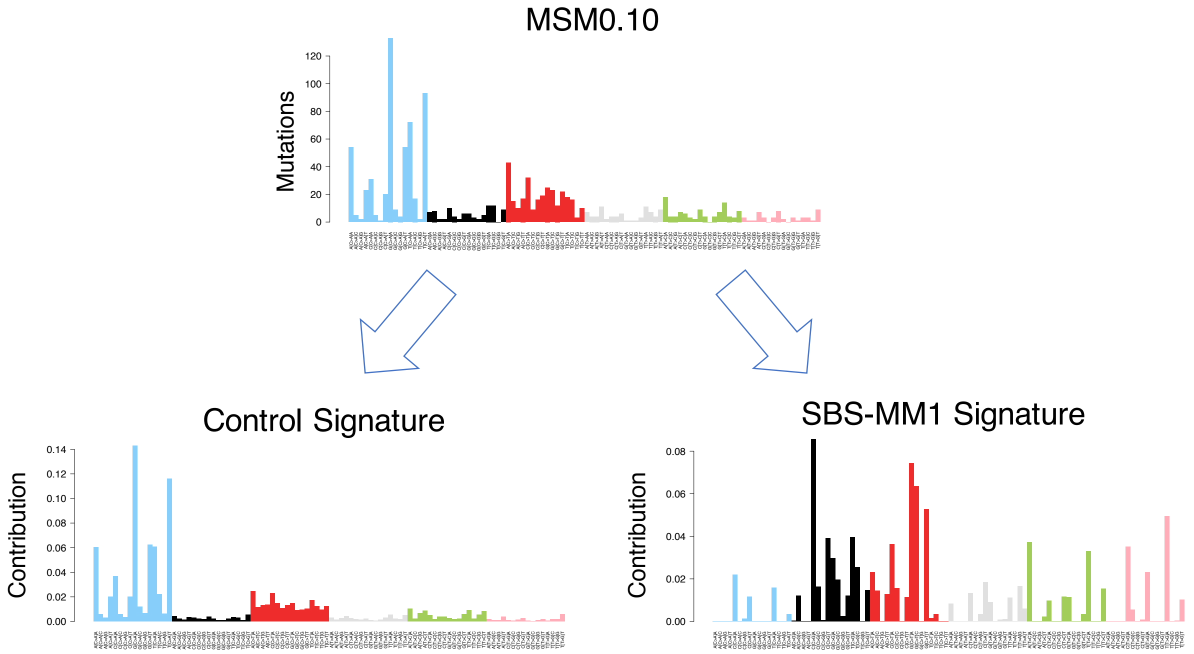

Supplementary Figure 4. Mutational signature contributions in human pluripotent stem cell lines. SBS-MM1 was absent in fifteen control cell lines who were not exposed to melphalan, and present in the only cell line that was exposed to melphalan (MSM0.10). The contribution of each signature was estimated using *mmsig*.

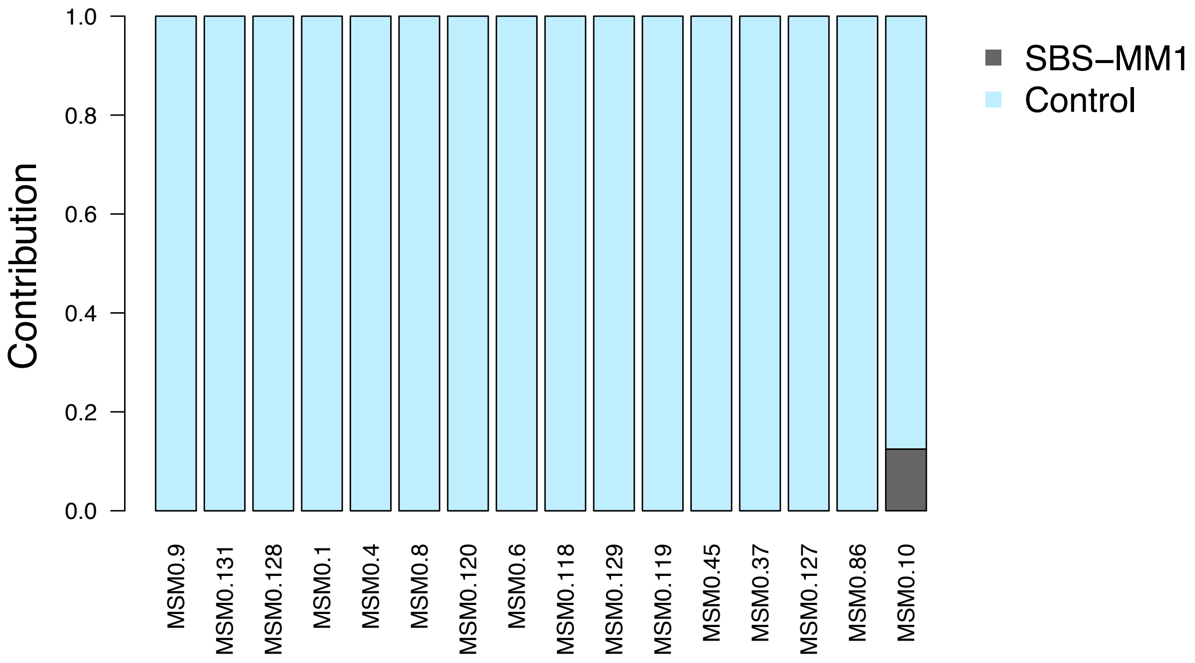

Supplementary Figure 5. Topography of mutational signatures in multiple myeloma. A) mutational signature contribution across the genome. Colored dots reflect chromosomal segments significantly enriched for distinct mutational processes using a negative binomial regression test. B) Example of the negative correlation between chromatin accessibility and mutation rate. C) Pairwise Pearson correlation between genomic features and mutational signature distribution across the genome. Point size and color intensity corresponds to the strength of positive (blue) or negative (red) correlation.

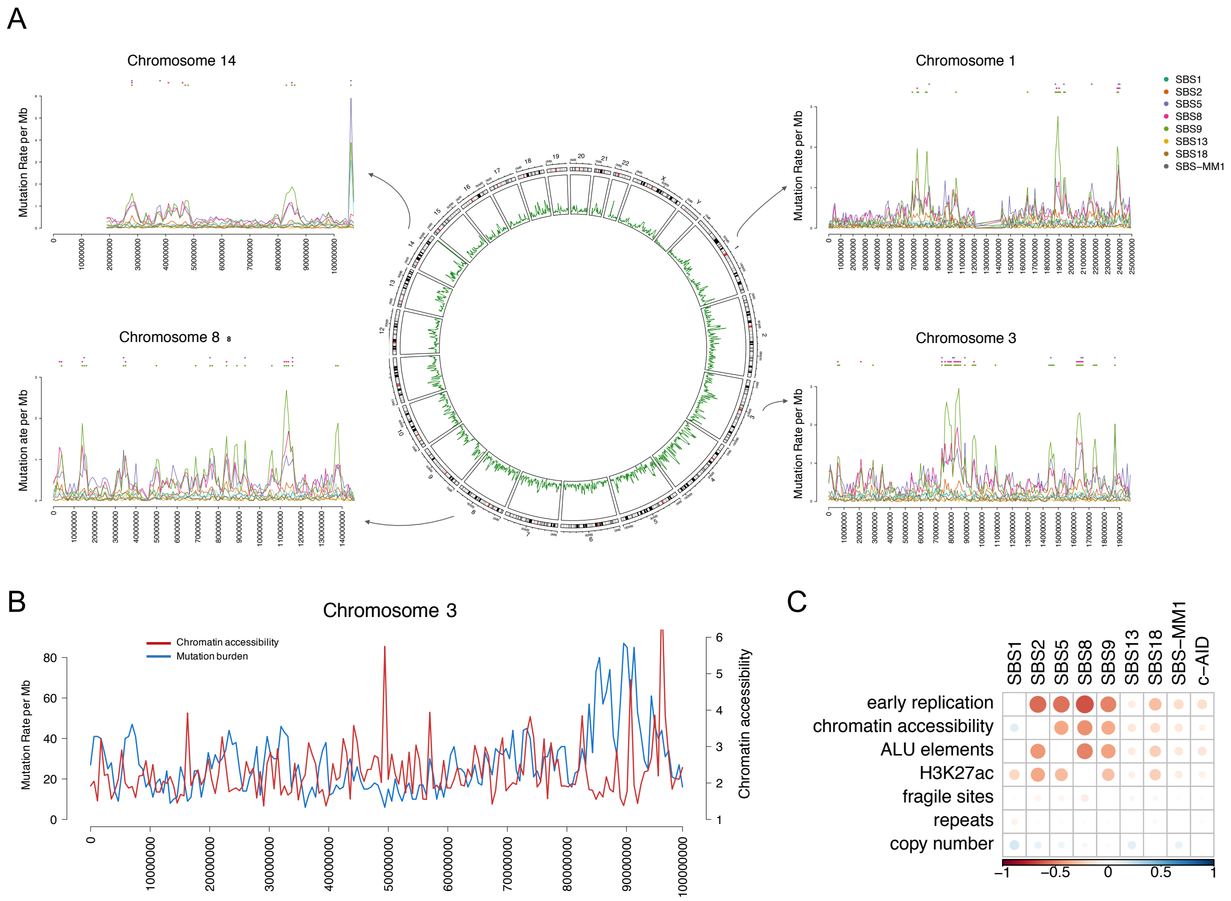

Supplementary Figure 6. 96-classes profile and mutational signature contribution of all mutations detected by WGS in the immunoglobulin loci of 52 MM patients. Confirming previous reports, the main active mutational processes in these regions were nc-AID and c-AID.

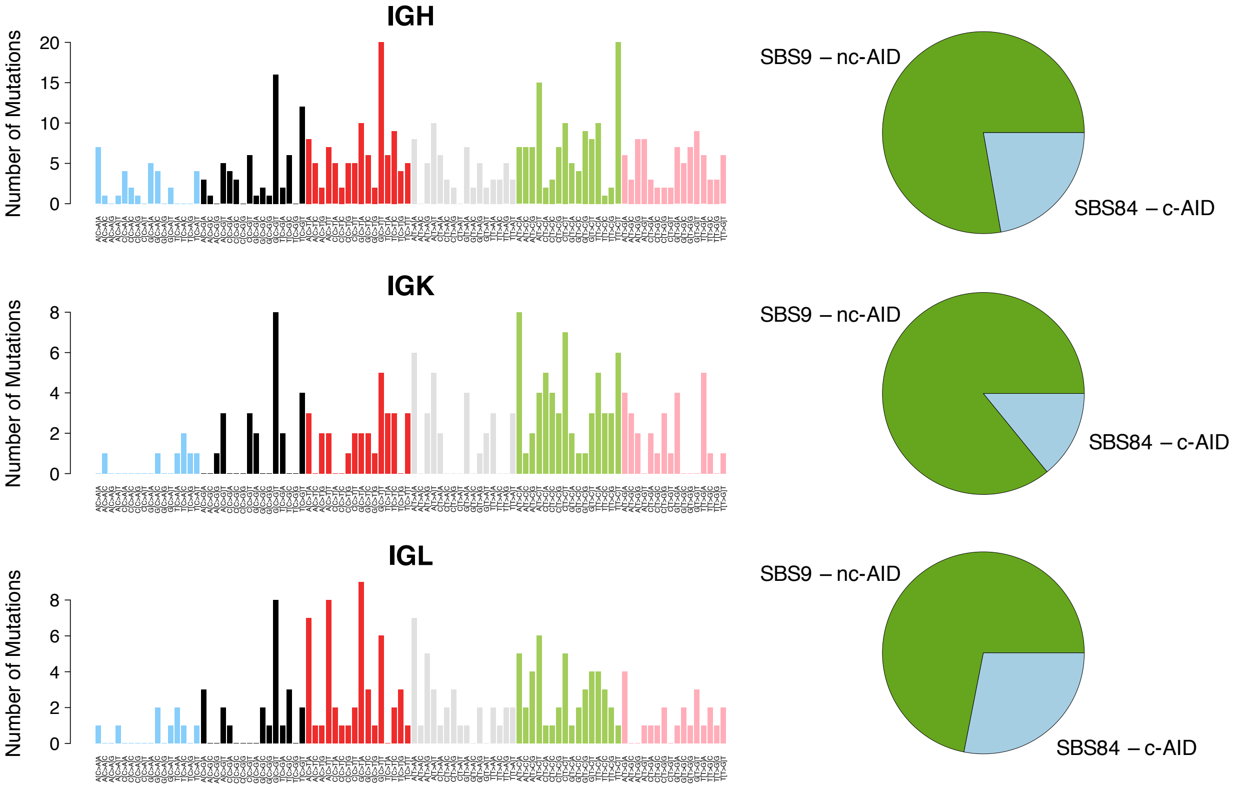

Supplementary Figure 7. Early and late c-AID activity in multiple myeloma. A) 96 classes and mutational signature contribution of mutations in the IGH locus that were not identified in all samples from the same patient by WGS (n = 26 patients). B) Similar analysis as in A, for patients with whole exome sequencing and multiple samples in the CoMMpass dataset (n=72). C) Number of mutations identified in the IGH locus of 72 patients with sequential samples in the CoMMpass dataset. For each patient (points), mutation frequencies are presented for mutations shared across all samples, uniquely identified at baseline and uniquely identified at relapse. D) Expression data showing that *AICDA* (the gene encoding AID) is not expressed in MM (n = 792 patients). For comparison, *APOBEC3A*, *APOBEC3B* and *CCND1* expression are also shown. Each point represents a sample and is colored by disease status.

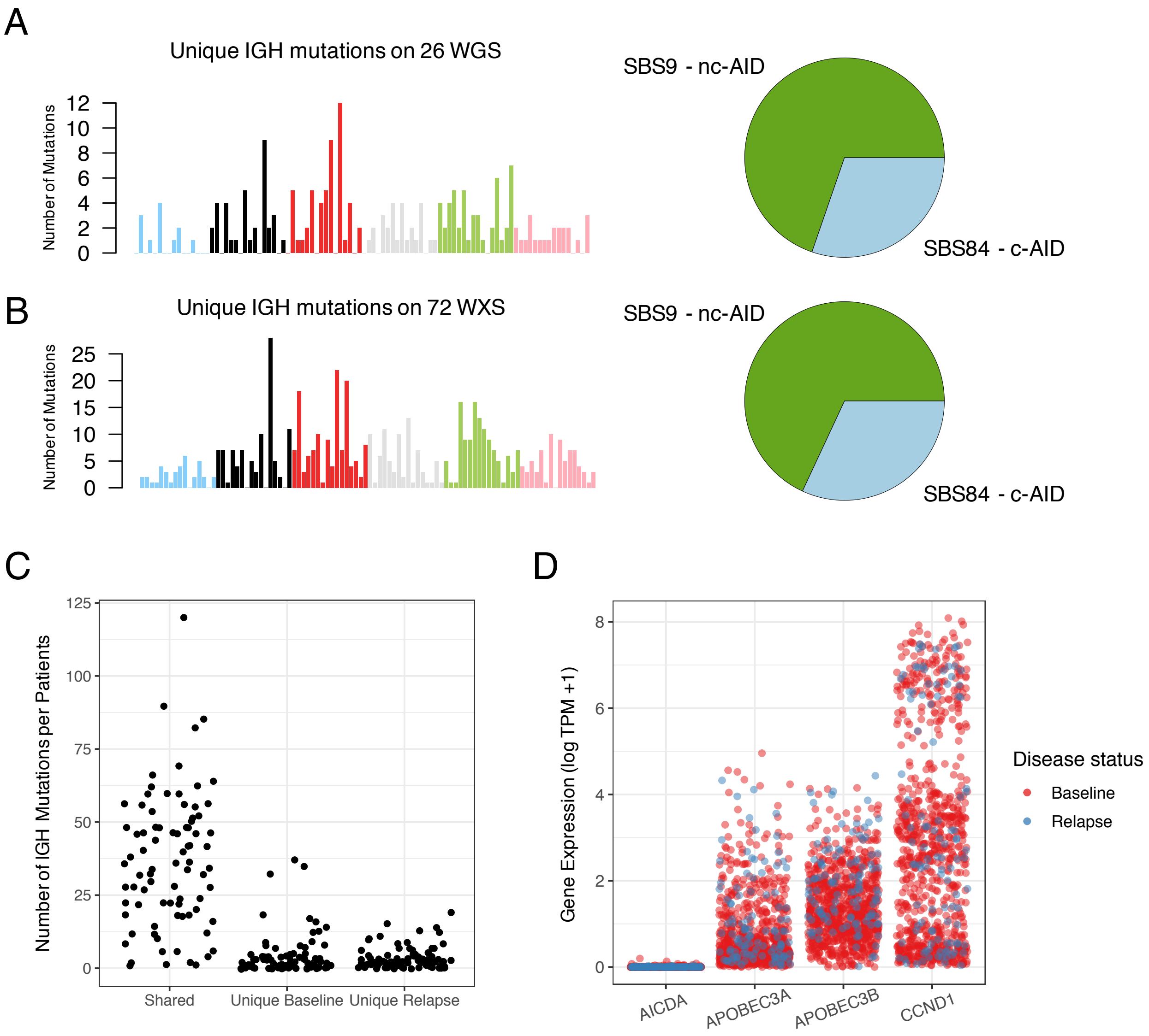

A
Unique IGH mutations on 26 WGS
Number of Mutations
0
2
4
6
8
10
12
SBS9 - nc-AID
SBS84 - c-AID
B
Unique IGH mutations on 72 WXS
Number of Mutations
0
5
10
15
20
25
SBS9 - nc-AID
SBS84 - c-AID
C
Number of IGH Mutations per Patients
0
25
50
75
100
125
Shared
Unique Baseline
Unique Relapse
D
Gene Expression (log TPM +1)
0
2
4
6
8
AICDA
APOBEC3A
APOBEC3B
CCND1
Disease status
Baseline
Relapse

Supplementary Figure 8. Transcriptional strand bias profile of the trunk and main branches from patients with multiple samples and evidence of SBS-MM1. All but one clusters of late clonal/subclonal mutations (PD26414 - D) were characterized by strong transcriptional strand bias in distinct C>T trinucleotide contexts.

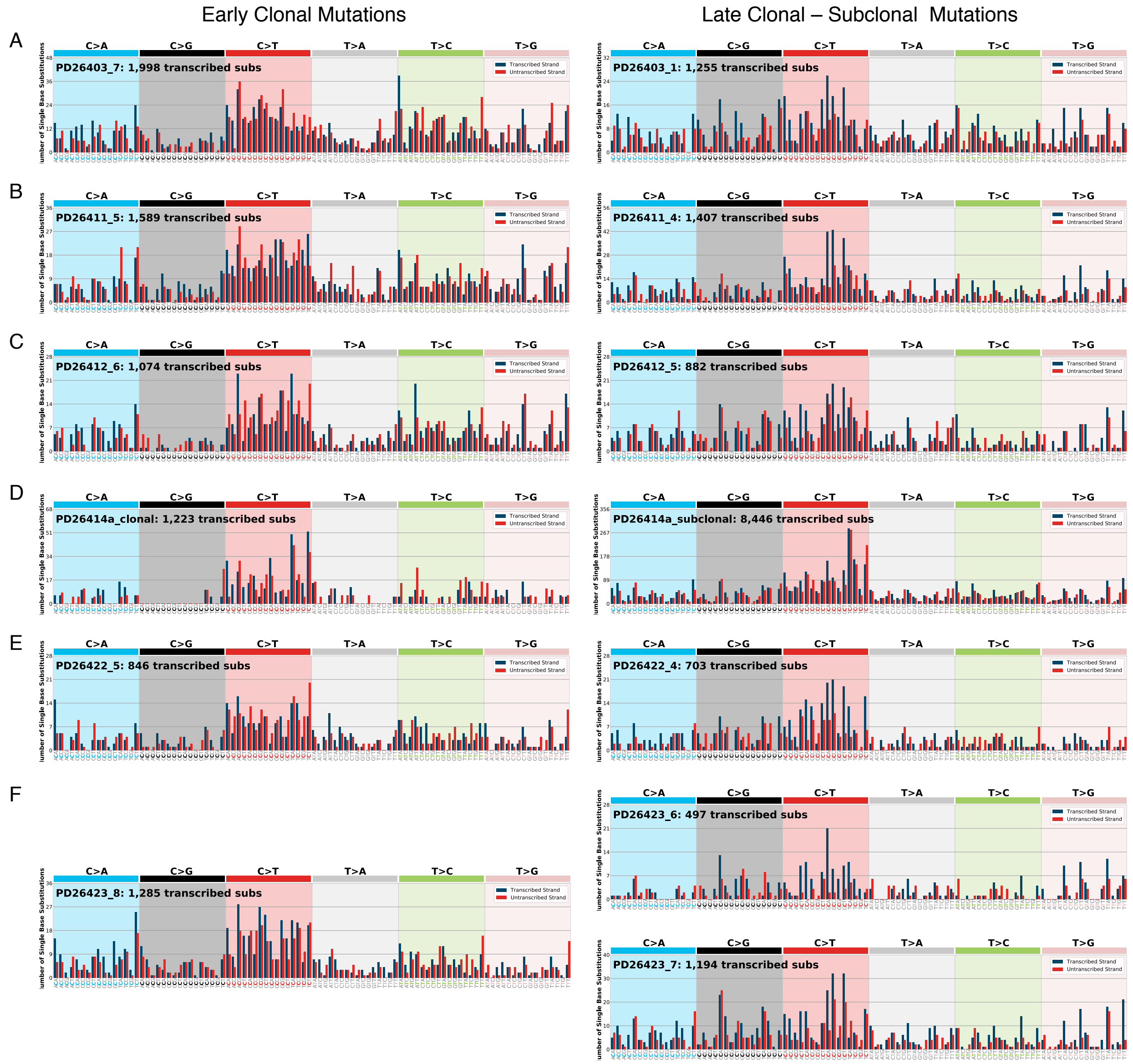

Supplementary Figure 9. Patient PD26403 was an emblematic example of the therapy-related nature of SBS-MM1. From this patient we analyzed three samples: one collected at the smoldering phase, one during the progression into symptomatic MM and one at first relapse after therapy with high dose melphalan (A). SBS-MM1 was absent in the first two samples but emerged as the main mutational process in the subclone that became dominant at relapse (B-D). B) Two-dimensional density plots showing the Dirichlet process clustering of the fraction of tumor cells carrying each mutation at each time point; on x-axis and y axis are plotted the different phases: smoldering multiple myeloma (SMM), newly diagnosed multiple myeloma (NDMM) and relapsed multiple myeloma (MM RR). Increasing intensity of red indicates the location of a high posterior probability of a cluster. C) Phylogenetic trees generated from the Dirichlet process analysis. The trunk (#1) and branch (#2 - #4) lengths are proportional to the total (sub)clone mutational load. D) Pie plot showing the mutational signature profile in the trunk and branches.

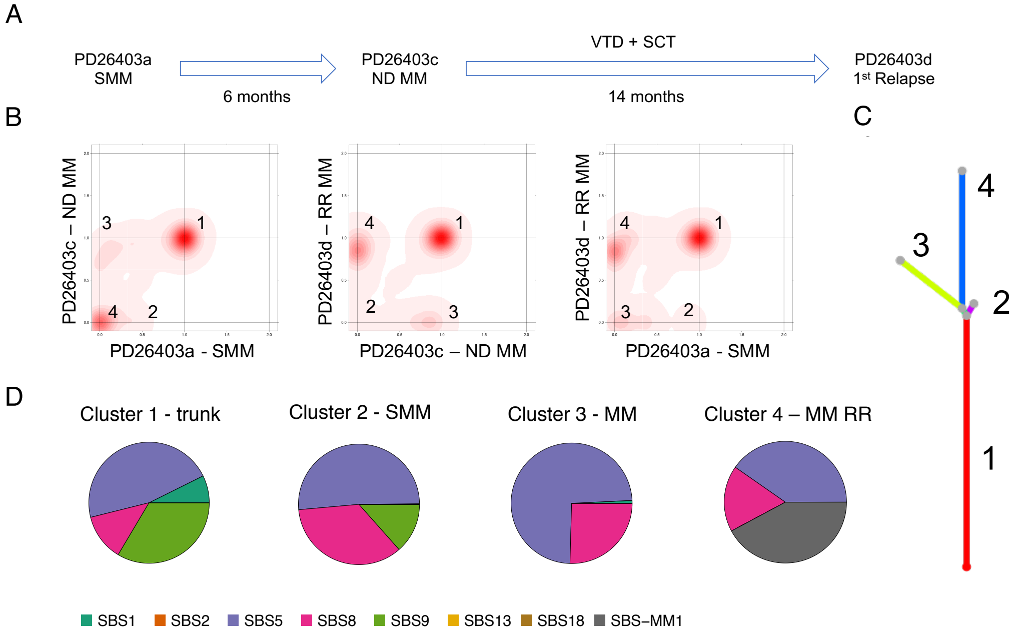

Supplementary Figure 10. AID activity in APOBEC hypermutated patients. 96-mutational profile of the immunoglobulin loci of two WGS cases with MAFA/MAFB (A), for WXS cases enrolled within the CoMMpass trial with (B) and without (C) high APOBEC activity.

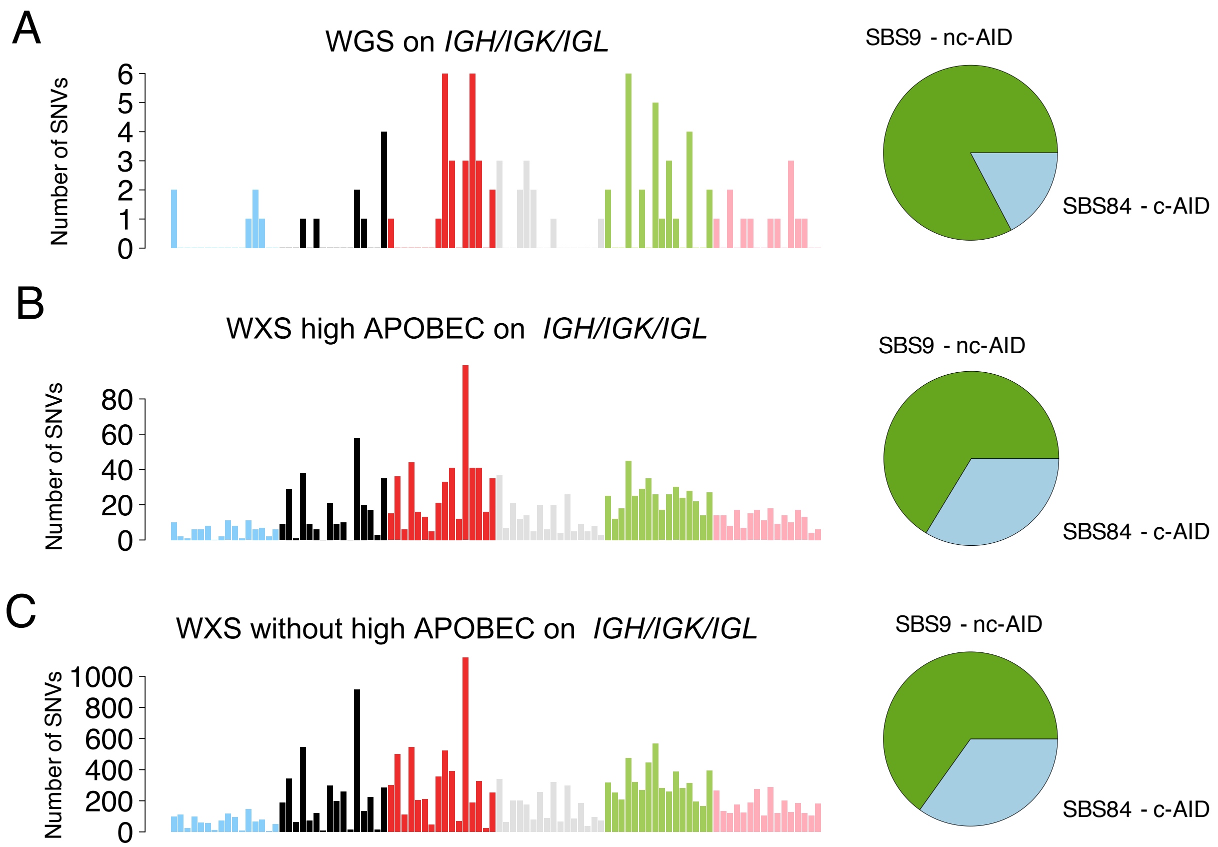

Supplementary Figure 11. SBS5 mutation rate (mutations per year) of each multiple myeloma patient included in the molecular clock analysis, as determined by our final linear mixed effect (LME) model (see also Supplementary Data 3). The average SBS5 mutation rate is shown as a horizontal black line. Dots (i.e. mutation rate estimates) with error-bars (standard deviation) were colored by the disease stage: red for patients who entered the study as smoldering multiple myeloma and then progressed; blue for patients who only had the earliest sample with newly diagnosed myeloma; and green for patients who relapsed.

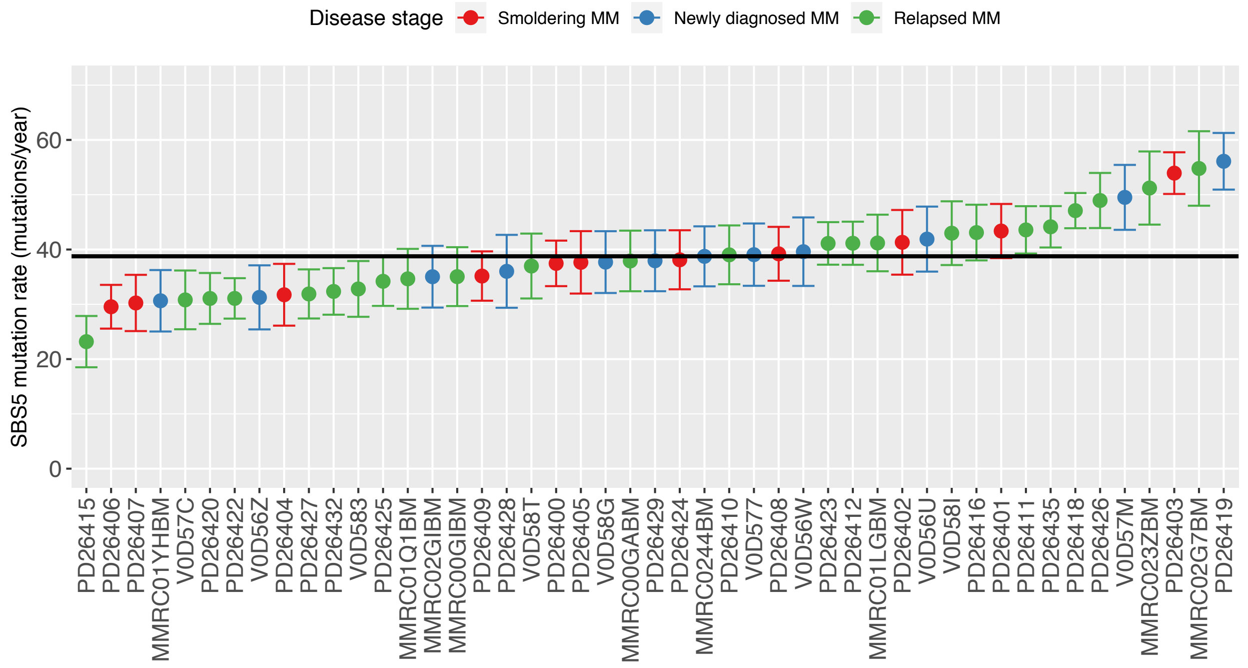

Supplementary Figure 12. Linear mixed effect (LME) models to define SBS5 mutation rate in multiple myeloma, to serve as a molecular clock. Comparing the predicted mutation counts from two LME models (simple and adjusted) with observed mutation counts for each phylogenetic branch. The simple LME model was based on patient age as the only covariate. The adjusted model also included several potential confounders: sample purity, ploidy and sequencing coverage, as well as includes a quadratic term for age (allowing the mutation rate to increase parabolically with time). The plot panels show the adjusted vs. simple LME models (left); observed vs simple model (center); and observed vs. adjusted (right). Black lines are drawn along the diagonals, i.e. where points are expected to cluster if the x and y variables are identical. Axis labels for LME models indicate the formulae used to fit the model (see also Supplementary Data 3).

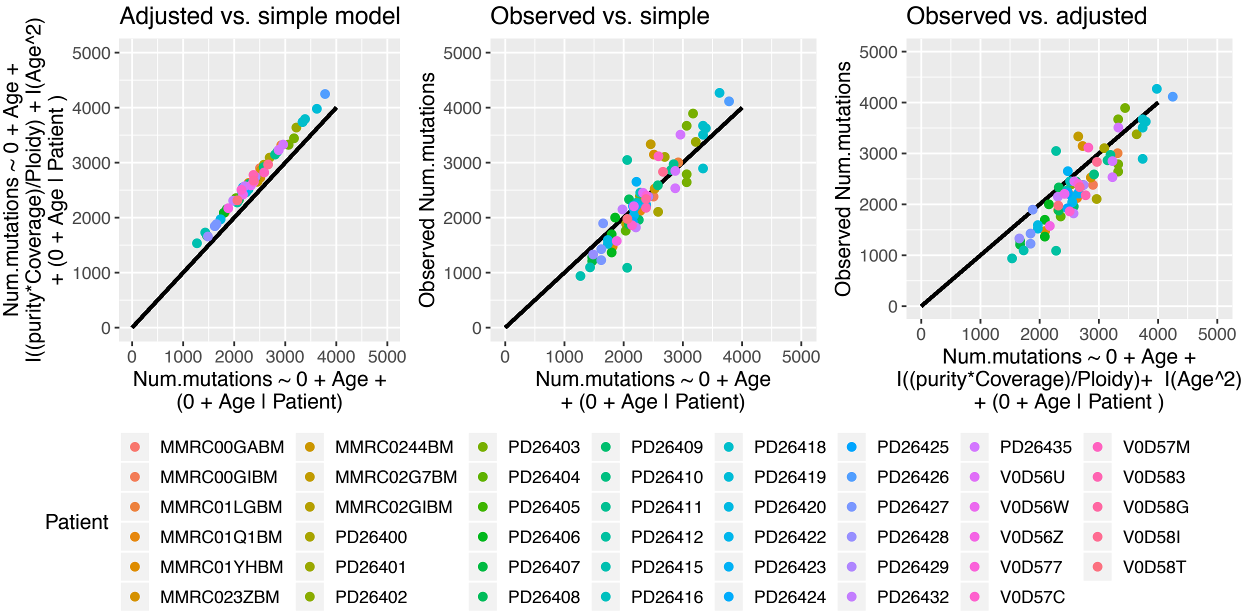

Supplementary Figure 13. Timing the most common recent ancestor (MRCA) in multiple myeloma. Blue dots and lines represent the MRCA estimates and 95% CI for each patient (see also Supplementary Data 3).

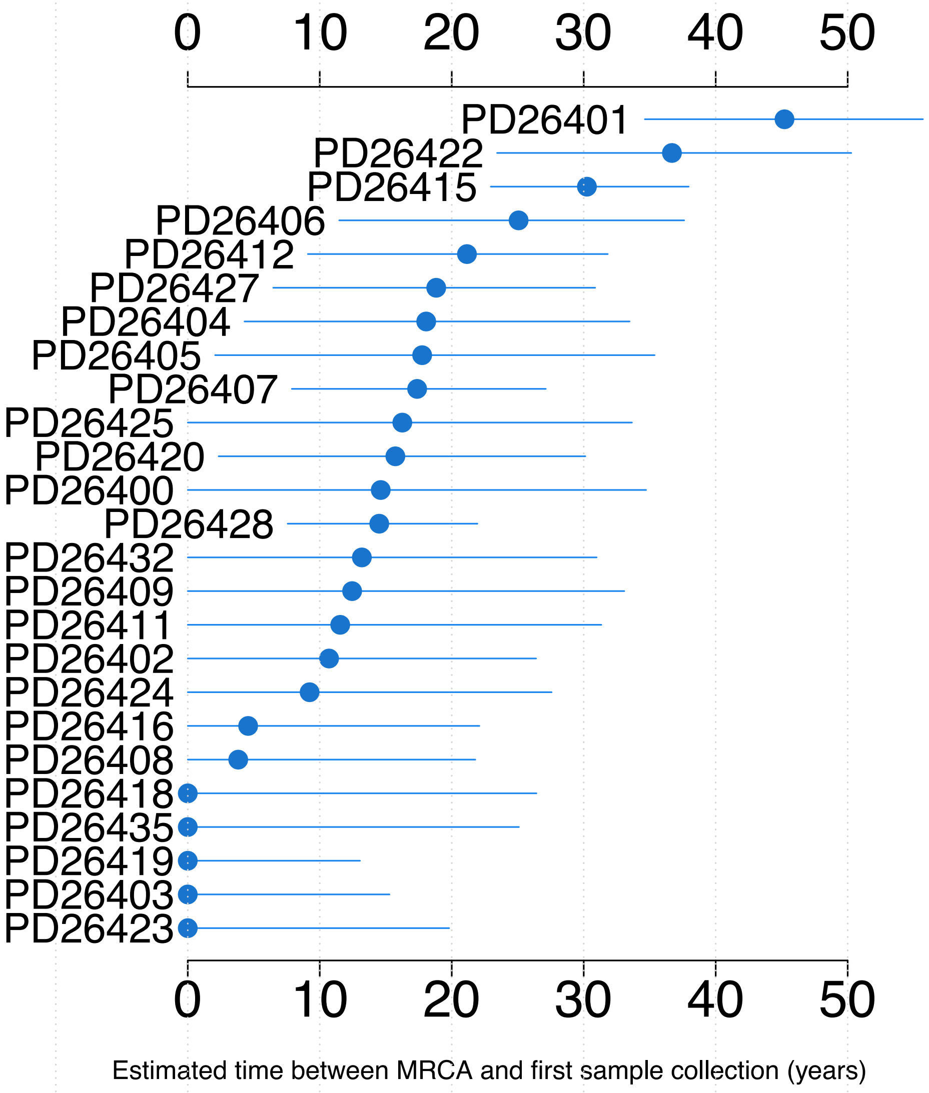

Supplementary Figure 14. Impact of uncertainty in mutational signature fitting on timing of landmark events. To account for the uncertainty in mutational signature fitting, we show here the timing estimates for the first multi-gain event based on the 2.5th and 97.5th percentiles of SBS5 mutational burden. Dots represent the point estimate for patient age, with error bars representing the 95 % confidence interval (see also Supplementary Data 3). A dashed black horizontal line is drawn at age thirty years to illustrate the majority of multi-gain events before this time-point.

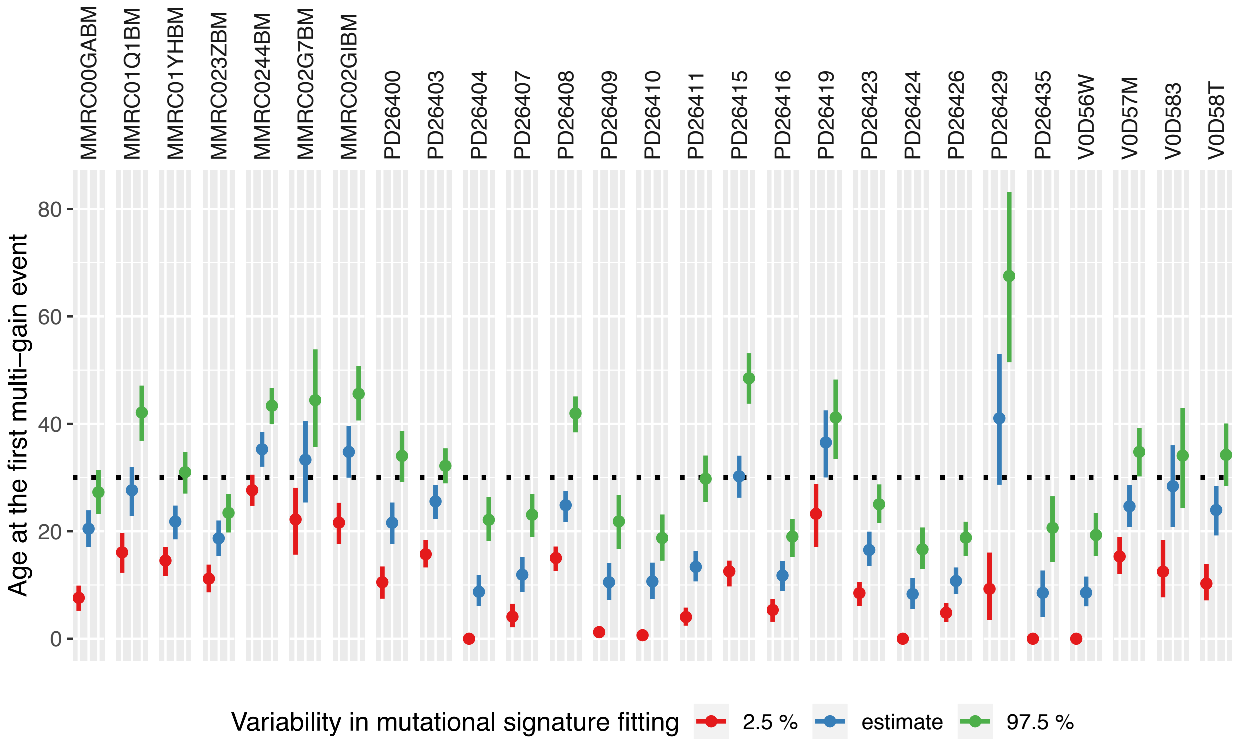

SUPPLEMENTARY TABLES

Supplementary Table 1. A summary of the treatments for patients with multiple samples collected at different time points

Sample ID	Status	Sampling Date	Treatment before collection	Post-SCT
**PD26400a**	SMM	19/08/2008	-	-
**PD26400c**	MM DG	12/01/2012	-	-
**PD26401a**	SMM	30/03/2010	-	-
**PD26401c**	MM DG	06/10/2010	-	-
**PD26402a**	SMM	20/04/2010	-	-
**PD26402c**	MM DG	20/02/2012	-	-
**PD26403a**	SMM	21/04/2010	-	-
**PD26403c**	MM DG	19/10/2010	-	-
**PD26403d**	MM RR	29/12/2011	Bortezomib-Thalidomide-Dexamethasone + SCT	yes
**PD26404a**	SMM	09/09/2010	-	-
**PD26404c**	MM DG	23/02/2011	-	-
**PD26405a**	SMM	03/11/2010	-	-
**PD26405c**	MM DG	18/01/2011	-	-
**PD26406a**	SMM	18/01/2010	-	-
**PD26406c**	MM DG	17/01/2012	-	-
**PD26407a**	SMM	23/02/2011	-	-
**PD26407c**	MM DG	12/10/2011	-	-
**PD26408a**	SMM	26/10/2011	-	-
**PD26408c**	MM DG	26/12/2011	-	-
**PD26409a**	SMM	21/03/2012	-	-
**PD26409c**	MM DG	28/01/2014	-	-
**PD26411c**	MM RR	18/10/2006	Cycles Bortezomib-Tanespimycin	yes
**PD26411d**	MM RR	24/01/2007	Cycles Bortezomib-Tanespimycin	yes
**PD26411a**	MM RR	17/04/2007	Bortezomib-Perifosine	yes
**PD26412a**	MM RR	12/02/2008	Marizomib	yes
**PD26412c**	MM RR	19/10/2011	Elotuzumab-Lenalidomide-Dexamethasone	yes
**PD26412d**	MM RR	11/11/2011	Elotuzumab-Lenalidomide-Dexamethasone	yes
**PD26414a**	MM RR	06/03/2007	Bortezomib-Cyclophosphamide-Thalidomide-Dexamethasone	no
**PD26414b**	MM RR	13/11/2008	Bortezomib-Cyclophosphamide-Thalidomide-Dexamethasone	no
**PD26414e**	MM RR	21/09/2010	Vorinostat- Lenalidomide-Dexamethasone	no
**PD26414f**	MM RR	07/12/2010	Bortezomib-CXCR4 inhibitor 3 cycles	no
**PD26414g**	MM RR	07/03/2011	Melphalan-Dexamethasone	no
**PD26415c**	MM RR	09/01/2006	Tanespimycin	no
**PD26415g**	MM RR	11/04/2013	Bortezomib- Tanespimycin	yes
**PD26416d**	MM RR	08/07/2008	Bortezomib-Perifosine	no
**PD26416e**	MM RR	21/04/2009	Bortezomib-Enzastaurin	no
**PD26418a**	MM RR	25/10/2007	Bortezomib-Lenalidomide-Dexamethasone	no
**PD26418c**	MM RR	23/01/2008	Bortezomib-Lenalidomide-Dexamethasone- Cyclophosphamide	no

**PD26418d**	MM RR	06/04/2009	Bortezomib-Lenalidomide-Dexamethasone - Cyclophosphamide and SCT	yes
**PD26418e**	MM RR	01/09/2009	DCEP-Thalidomide-Bortezomib	yes
**PD26419a**	MM DG	14/03/2005	-	no
**PD26419c**	MM RR	30/07/2007	Bortezomib-Thalidomide-Dexamethasone- Perifosine	no
**PD26419d**	MM RR	08/07/2008	bortezomib-cyclophosphamide-thalidomide-dexamethasone (12 cycles)	no
**PD26420a**	MM RR	01/01/2007	Thalidomide–Dexamethasone and Bortexomib-Doxorubicin–Dexamethasone, and Melphalan/Dexamethasone	yes
**PD26420c**	MM RR	18/05/2007	SGN40	yes
**PD26422d**	MM RR	11/10/2007	10 Bortezomib-Lenalidomide-dexamethasone and DCEP and 2nd SCT	yes
**PD26422e**	MM RR	24/05/2007	8 cycles Aplidin	yes
**PD26422f**	MM RR	08/04/2008	8 cycles Bortezomib + Perifosine	yes
**PD26423e**	MM RR	08/04/2008	Bortezomib-Dexamethasone	yes
**PD26423g**	MM RR	29/06/2011	Bortezomib-Lenalidomide-Dexamethasone	yes
**PD26423h**	MM RR	14/12/2011	Bortezomib/LY2127399	yes
**PD26424a**	SMM	21/10/2010	-	-
**PD26424c**	MM RR	20/10/2011	Lenalidomide-Dexamethasone	no
**PD26425e**	MM RR	01/07/2008	Bortezomib-mTor Inhibitor	yes
**PD26425f**	MM RR	09/01/2009	SGN40-Lenalidomide	yes
**PD26427a**	MM DG	19/09/2006	-	-
**PD26427c**	MM RR	15/03/2007	Bortezomib-Lenalidomide-Dexamethasone	no
**PD26428a**	MM DG	08/01/2007	-	-
**PD26428c**	MM RR	17/06/2008	Bortezomib-Lenalidomide-Dexamethasone	no
**PD26432c**	MM RR	09/09/2008	Bortezomib-Lenalidomide-Dexamethasone	no
**PD26432e**	MM RR	02/12/2014	Bortezomib Maintenance	no
**PD26435c**	MM RR	14/01/2008	Bortezomib-Dexamethasone+Hsp90 Inhibitor	no
**PD26435e**	MM RR	16/06/2010	Pomalidomide-Dexamethasone	no

SCT: stem cell transplantation.
SMM: smoldering multiple myeloma
MM DG: multiple myeloma at diagnosis
MM RR: multiple myeloma at relapse

Supplementary Table 2. Association between SBS-MM1 and clinical and genomic features present in more than 5 patients. p values were generated using Fisher's exact test and corrected by false rate discovery.

Feature	p-value	q-value
**Age>65 years**	0.69939486	0.89271746
**Gender**	0.7061695	0.89271746
**Stage**	0.00796536	0.06637798
**Stem cell transplant**	0.0044252	0.05531494
**Cancer Cell Fraction**	0.64540562	0.89271746
**t(11;14)**	1	1
**t(4;14)**	1	1
**translocation on MYC**	0.71417397	0.89271746
**HRD**	0.26828536	0.60973945
**del13q34**	0.14413334	0.4504167
**del1p22**	0.18518257	0.50722215
**del20q13**	1	1
**del6q25**	1	1
**del8p22**	0.132658	0.4504167
**del8q24**	1	1
**delCDKN2C**	0.05677839	0.23657663
**delCYLD**	0.04321802	0.21609011
**delFAM46C**	0.20288886	0.50722215
**delRB1**	0.46897908	0.83746264
**delTP53**	0.64527049	0.89271746
**delTRAF3**	0.57445915	0.89271746
**gain1q21**	0.02485249	0.15532809
**KRAS**	0.32995537	0.67154665
**NRAS**	0.34920426	0.67154665

Supplementary Table 3. VJ and HCDR amino acid sequence of 26 MM patients with more one sample collected at different time points.

Sample	V	J	CDR3
**PD26400a**	IGHV4-61	IGHJ4	CARDPERHPDMFCTGDNCYPSW
**PD26400c**	IGHV4-59	IGHJ4	CARDPERHPDMFCTGDNCYPSW
**PD26401a**	IGHV3-11	IGHJ6	CATVSREGLGTNYYYYMDVW
**PD26401c**	IGHV3-11	IGHJ6	CATVSREGLGTNYYYYMDVW
**PD26402a**	IGHV4-39	IGHJ2	CAGPPTNEWYFALW
**PD26402c**	IGHV4-39	IGHJ2	CAGPPTNEWYFALW
**PD26403a**	None	None	None
**PD26403c**	None	None	None
**PD26403d**	None	None	None
**PD26404a**	IGHV3-9	IGHJ3	CAKDLFRGYSYGYWGAFDLW
**PD26404c**	IGHV3-9	IGHJ3	CAKDLFRGYSYGYWGAFDLW
**PD26405a**	IGHV3-74	IGHJ6	CCRYSSYWTFRYYGVDVW
**PD26405c**	IGHV3-74	IGHJ6	CCRYSSYWTFRYYGVDVW
**PD26406a**	IGHV3-11	IGHJ4	CVRTHFGTEQWEVLFDHW
**PD26406c**	IGHV3-11	IGHJ4	CVRTHFGTEQWEVLFDHW
**PD26407a**	IGHV3-30	IGHJ6	None
**PD26407c**	IGHV3-30	IGHJ6	CAKDAFESGSDREYRDYSHYYGLDVW
**PD26408a**	IGHV3-9	IGHJ6	CAKASGQLELAGYYYYYPMDVW
**PD26408c**	IGHV3-9	IGHJ6	CAKASGQLELAGYYYYYPMDVW
**PD26409a**	IGHV1-18	IGHJ1	CARDLSIGVTGTESFQHW
**PD26409c**	IGHV1-18	IGHJ1	CARDLSIGVTGTESFQHW
**PD26411a**	IGHV3-7	IGHJ4	CARGSNYDFW
**PD26411b**	None	None	None
**PD26411c**	IGHV4-39	IGHJ6	CARQVGSSSFLYYYYYMDVW
**PD26411d**	IGHV3-7	IGHJ4	CARGSNYDFW
**PD26412a**	None	None	None
**PD26412c**	IGHV4-31	IGHJ4	None
**PD26412d**	IGHV4-28	IGHJ5	None
**PD26414a**	IGHV5-51	IGHJ4/IGHJ5	None
**PD26414b**	IGHV5-51	IGHJ4	None
**PD26414c**	IGHV5-51	IGHJ5	None

**PD26414e**	IGHV5-51	IGHJ5	None
**PD26414f**	IGHV5-51	IGHJ4	None
**PD26414g**	IGHV5-51	IGHJ4/IGHJ5	None
**PD26415c**	None	None	None
**PD26415g**	IGHV3-30	IGHJ6	None
**PD26416d**	IGHV5-51	IGHJ2	CARAHGYNAFYWYFDLW
**PD26416e**	IGHV5-51	IGHJ2	CARAHGYNAFYWYFDLW
**PD26418a**	IGHV3-15	IGHJ4	CANSEVGAKPIDYW
**PD26418c**	IGHV3-15	IGHJ5	CANSEVGAKPIDYW
**PD26418d**	IGHV3-15	IGHJ5	CANSEVGAKPIDYW
**PD26418e**	IGHV3-15	IGHJ5	CANSEVGAKPIDYW
**PD26419a**	IGHV1-69	IGHJ4	CARAPNGYSNLFDSW
**PD26419c**	IGHV1-69	IGHJ4	CARAPNGYSNLFDSW
**PD26419d**	IGHV1-69	IGHJ4	CARAPNGYSNLFDSW
**PD26420a**	IGHV3-33	IGHJ4	CVRLQGVGAYQQLDLW
**PD26420c**	IGHV3-33	IGHJ4	CVRLQGVGAYQQLDLW
**PD26422d**	IGHV2-70	IGHJ5	CARGSGAIDYW
**PD26422e**	IGHV2-70	IGHJ4	None
**PD26422f**	IGHV2-70	IGHJ5	CARGSGAIDYWSQGTLVTVSSGESSPPPL
**PD26423e**	IGHV4-31	IGHJ6	CARSYYNGFYHSYYNGLDVW
**PD26423g**	IGHV4-31	IGHJ6	CARSYYNGFYHSYYNGLDVW
**PD26423h**	IGHV4-31	IGHJ3	CARSYYNGFYHSYYNGLDVW
**PD26424a**	None	None	None
**PD26424c**	None	None	None
**PD26425e**	IGHV3-21	IGHJ4	CARDKKTGWYNGAGYW
**PD26425f**	IGHV3-21	IGHJ4	CARDKKTGWYNGAGYW
**PD26427a**	IGHV3-13	IGHJ6	None
**PD26427c**	IGHV3-13	IGHJ6	None
**PD26428a**	IGHV2-5	IGHJ4	CARTVRGAVPFDFW
**PD26428c**	IGHV2-5	IGHJ4	CARTVRGAVPFDFW
**PD26435c**	IGHV4-39	IGHJ4	CVRDTYSYDRSGYGILDYW
**PD26435e**	IGHV4-39	IGHJ4	CVRDTYSYDRSGYGILDYW

SUPPLEMENTARY REFERENCES

1 Maura, F. *et al.* A practical guide for mutational signature analysis in hematological malignancies. *Nature communications* **10**, 2969, doi:10.1038/s41467-019-11037-8 (2019).

2 Kucab, J. E. *et al.* A Compendium of Mutational Signatures of Environmental Agents. *Cell* **177**, 821-836.e816, doi:10.1016/j.cell.2019.03.001 (2019).
