# Supplementary material for: Timing the initiation of multiple myeloma
Source: Nat Commun. 2020 Apr 21;11:1917. doi: 10.1038/s41467-020-15740-9 (PMC7174344; doi:10.1038/s41467-020-15740-9)
Supplement: Supplementary file 3 — Description of Additional Supplementary Information [file 41467_2020_15740_MOESM3_ESM.pdf]

Description of Additional Supplementary Files

File Name: Supplementary Data 1
Description: Clinical and biological characterization of 52 multiple myeloma patients included in this study.

File Name: Supplementary Data 2
Description: Mutational signature profile of each patient.

File Name: Supplementary Data 3
Description: Transcriptional strand bias comparison between patient with and without SBS-MM1.

File Name: Supplementary Data 4
Description: Transcriptional strand bias for SBS-MM1 signficant trinucleotide context for each patient. p value were generated by mmsig applying poisson test.

File Name: Supplementary Data 5
Description: Assignment of signatures for whole-genome sequencing (WGS) data of one human-induced pluripotent stem cells exposed to melphalan (MSM0.10) and 15 controls (Kucab, et al Cell 2019). Each of the signatures included in the COSMIC-49 catalogue were tested. Cosine similarities between the extracted all single and all combination of signatures included in the COSMIC-49 catalogue were computed.

File Name: Supplementary Data 6
Description: R code used to explain the 96-class mutational profile of human-induced pluripotent stem cell clones exposed to melphalan (Kucab et al. Cell 2019).

File Name: Supplementary Data 7
Description: Clinical and biological characterization of 24 multiple myeloma patients included in the CoMMpass study with samples collected at baseline and first relapse.

File Name: Supplementary Data 8
Description: R code used to identify clock-like mutational process in the CoMMpass data set (764 patients with available whole exome sequencing data).

File Name: Supplementary Data 9
Description: R code used to estimate the mutation rate and to time the first multi-gain events in multiple myeloma.
