# Supplementary material for: Timing the initiation of multiple myeloma
Source: Nat Commun. 2020 Apr 21;11:1917. doi: 10.1038/s41467-020-15740-9 (PMC7174344; doi:10.1038/s41467-020-15740-9)
Supplement: Supplementary file 9 — Supplementary Data 6 [file 41467_2020_15740_MOESM9_ESM.html]

Explaining the 96-class mutational profile of human-induced pluripotent stem cell clones exposed to melphalan


# Explaining the 96-class mutational profile of human-induced pluripotent stem cell clones exposed to melphalan

## Introduction

Kucab et al Cell 2019 has recently reported a comprehensive catalog of distinct mutational signatures induced by exposing human-induced pluripotent stem cells (iPSC) to different exogenous exposures such as chemotherapeutic agents. Melphalan was tested in one single cell expansion experiment, but was not included in the final analysis because the “signal-to-noise-ratio” was <2. Here, we analyze these cell lines following the recently published workflow for “assignment” of mutational signatures (Maura et al. Nat comm 2019; doi.org/10.1038/s41467-019-11037-8).The cell line exposed to melphalan was MMS0.10; the remaining cell lines were grown in different control conditions.

## Setup

```
library(deconstructSigs)
library(TxDb.Hsapiens.UCSC.hg19.knownGene)
```

```
## Loading required package: GenomicFeatures
```

```
## Warning: package 'GenomicFeatures' was built under R version 3.5.3
```

```
## Loading required package: BiocGenerics
```

```
## Loading required package: parallel
```

```
## 
## Attaching package: 'BiocGenerics'
```

```
## The following objects are masked from 'package:parallel':
## 
##     clusterApply, clusterApplyLB, clusterCall, clusterEvalQ,
##     clusterExport, clusterMap, parApply, parCapply, parLapply,
##     parLapplyLB, parRapply, parSapply, parSapplyLB
```

```
## The following objects are masked from 'package:stats':
## 
##     IQR, mad, sd, var, xtabs
```

```
## The following objects are masked from 'package:base':
## 
##     anyDuplicated, append, as.data.frame, basename, cbind, colMeans,
##     colnames, colSums, dirname, do.call, duplicated, eval, evalq,
##     Filter, Find, get, grep, grepl, intersect, is.unsorted, lapply,
##     lengths, Map, mapply, match, mget, order, paste, pmax, pmax.int,
##     pmin, pmin.int, Position, rank, rbind, Reduce, rowMeans, rownames,
##     rowSums, sapply, setdiff, sort, table, tapply, union, unique,
##     unsplit, which, which.max, which.min
```

```
## Loading required package: S4Vectors
```

```
## Loading required package: stats4
```

```
## 
## Attaching package: 'S4Vectors'
```

```
## The following object is masked from 'package:base':
## 
##     expand.grid
```

```
## Loading required package: IRanges
```

```
## Loading required package: GenomeInfoDb
```

```
## Loading required package: GenomicRanges
```

```
## Loading required package: AnnotationDbi
```

```
## Loading required package: Biobase
```

```
## Welcome to Bioconductor
## 
##     Vignettes contain introductory material; view with
##     'browseVignettes()'. To cite Bioconductor, see
##     'citation("Biobase")', and for packages 'citation("pkgname")'.
```

```
ref_genome = "BSgenome.Hsapiens.UCSC.hg19"
library(ref_genome, character.only = TRUE)
```

```
## Loading required package: BSgenome
```

```
## Loading required package: Biostrings
```

```
## Loading required package: XVector
```

```
## 
## Attaching package: 'Biostrings'
```

```
## The following object is masked from 'package:base':
## 
##     strsplit
```

```
## Loading required package: rtracklayer
```

```
library(nnls)
library(stringr)
library(RColorBrewer)
cos.sim <- function(a, b){
  return( sum(a*b)/sqrt(sum(a^2)*sum(b^2)) )
}  

options(scipen = 999)
```

## Load single cell expansion mutational data

Load the Kucab et al Cell 2019 data: 1 human cell line exposed to melphalan and 15 controls.

```
## cell lines annotation
readme<- read.delim("README.txt", stringsAsFactors = F, skip=16)
readme$code<- rownames(readme)
ser_code<- readme$Decode.of.the.MSM.number.and.treatment.[c(grep("Control", readme$Decode.of.the.MSM.number.and.treatment.),
                                                            grep("Melphalan", readme$Decode.of.the.MSM.number.and.treatment.))]
readme2<- readme[readme$Decode.of.the.MSM.number.and.treatment. %in% ser_code,]

## cell lines mutational catalogue annotation
mut<- read.delim("denovo_subclone_subs_final.txt", stringsAsFactors = F)
mut_mel<- mut[mut$Sample.Name %in% readme2$code,]


## upload Control signature 
control<- read.delim("Mutagen53_sub_signature.txt", sep="\t", stringsAsFactors = F)

## upload SBS-MM1 signature
sig_ref <- read.csv("mm_signature_definitions.csv", stringsAsFactors = F, header=T)

## Upload 49-COSMIC

COSMIC30_subs_signatures_new <- read.table("cosmic_new_signatures.csv",
                                           sep = ",",header = TRUE,
                                           check.names = FALSE,stringsAsFactors = FALSE)

## add SBS-MM1 and Control signatures to the new reference 
COSMIC30_subs_signatures_new$SBS_MM1 <- sig_ref$MM1
COSMIC30_subs_signatures_new$Control <-   control$Control
COSMIC30_subs_signatures_new<- COSMIC30_subs_signatures_new[,-c(1:2)]
COSMIC30_subs_signatures_new[,1:ncol(COSMIC30_subs_signatures_new)]<- apply(COSMIC30_subs_signatures_new[,1:ncol(COSMIC30_subs_signatures_new)], 2, function(x){as.numeric(as.character(x))})
```

## Identify the optimal combination of mutational signatures to explain the 96-mutational profile of each single cell expansion

For each growth condition (i.e., melphalan vs. 15 controls), we compared the observed mutational profile with every possible linear combination of known mutational signatures (COSMIC49 + SBS-MM1) and the control signature. Observed and reconstructed mutational profiles were compared and ranked by cosine similarity.

```
mut_mel$chr<- paste0("chr", mut_mel$Chrom)
mut_mel$chr[mut_mel$chr=="chr23"]<-"chrX"
mut_mel$chr[mut_mel$chr=="chr24"]<-"chrY"
all_sig_96<- mut.to.sigs.input(mut.ref = mut_mel,
                               sample.id = "Sample.Name",
                               chr = "chr",
                               pos = "Pos",
                               ref = "Ref",
                               alt = "Alt",
                               bsg = BSgenome.Hsapiens.UCSC.hg19)


signatures<- t(all_sig_96)


sims2 <- list()

for(i in (1:ncol(signatures)))
{
sims2[[paste0("process_",rownames(all_sig_96)[i])]] <- data.frame()
#similarities to individual COSMIC49
cs <-apply(COSMIC30_subs_signatures_new, 2, function(x) cos.sim(x,signatures[,i]))
code_sig<- names(cs)
for (j in 1:length(cs)) {
  sims2[[paste0("process_",rownames(all_sig_96)[i])]] <- rbind(sims2[[paste0("process_",rownames(all_sig_96)[i])]],
                                         data.frame(list(sig=code_sig[j]),
                                                    cossim=cs[j],
                                                    proportions=paste0(code_sig[j],"=1"),stringsAsFactors = FALSE))
}
#similarities to COSMIC49 combinations
for (x in 1:(ncol(COSMIC30_subs_signatures_new)-1)) {
  for (y in (x+1):(ncol(COSMIC30_subs_signatures_new))) {
    a <- COSMIC30_subs_signatures_new[,c(x,y)]
    #fit a non negative least squares linear model. A linear combination of two COSMIC signatures x and y.
    r <- nnls(A = as.matrix(a),b = as.vector(signatures[,i]))
    coeffnorm <- r$x/sum(r$x)
    sims2[[paste0("process_",rownames(all_sig_96)[i])]] <- rbind(sims2[[paste0("process_",rownames(all_sig_96)[i])]],
                                           data.frame(list(sig=paste0(code_sig[x], "-",code_sig[y])),
                                                      cossim=cos.sim(as.vector(as.matrix(a) %*% r$x),as.vector(signatures[,i])),
                                                      proportions=paste0(code_sig[x],"=",sprintf("%.2f",coeffnorm[1]),";",code_sig[y],
                                                                         "=",sprintf("%.2f",coeffnorm[2])),stringsAsFactors = FALSE))
  }
}

}

Suppl_table_5<-do.call("cbind", sims2)
```

The optimal combination of signatures to explain the observed mutational profile in melphalan exposed cells consisted of 84 % control signature and 16 % SBS-MM1 (see below). This combinatin of signatures showed a cosine similarity of 0.985 with the observed data.

```
Suppl_table_5[which(Suppl_table_5$process_MSM0.10.cossim == max(Suppl_table_5$process_MSM0.10.cossim)),grepl("MSM0.10", names(Suppl_table_5))]
```

```
##       process_MSM0.10.sig process_MSM0.10.cossim process_MSM0.10.proportions
## 12345     SBS_MM1-Control              0.9849237   SBS_MM1=0.16;Control=0.84
```

## Display the highest scoring non-control signature for melphalan-exposed and control cell lines.

```
cos_sim <-Suppl_table_5[,seq(2,ncol(Suppl_table_5), by=3)]
rownames(cos_sim)<- Suppl_table_5$process_MSM0.9.sig

vec<- seq(2,ncol(Suppl_table_5), by=3)[1:16]
cos_sim_col<- list()
for(i in (1:length(vec)))
{
  cos_sim_col[[i]]<- unlist(c(max(Suppl_table_5[,vec[i]]),
                  Suppl_table_5[which(Suppl_table_5[,vec[i]]==max(Suppl_table_5[,vec[i]])),][,c(vec[i]-1,vec[i]+1)], colnames(Suppl_table_5)[vec[i]] ))
}

cos_sim_col2<- do.call("rbind", cos_sim_col)
cos_sim_col2<- as.data.frame.matrix(cos_sim_col2)
#cos_sim_col2
```

Below we plot the highest-scoring non-control signature for each cell line. The percentage of mutations explained by the signature is shown on the y-axis. Bars are colored by the mutational signatue. SBS-MM1 was part of the highest-scoring combination in melphalan exposed cell line (MSM0.10), but none of the control cell lines.

```
out <- str_split_fixed(( cos_sim_col2$process_MSM0.9.proportions),'=',3)
out2<- str_split_fixed(( out[,2]), '[;]', 2)
counts<- as.data.frame(cbind(out[,-2], out2))
counts$cell_line<- gsub(".cossim","",cos_sim_col2$V4)
counts$cell_line<- gsub("process_","",counts$cell_line)
counts<- counts[,c(1,3, 4,2,5)]
counts<- counts[order(counts$V3),]
color<- c(RColorBrewer::brewer.pal(8, "Set3"), RColorBrewer::brewer.pal(6, "Set3"))
counts$V1<- as.factor(counts$V1)
jColors <-data.frame(V1 = levels(counts$V1),
                   color = c(RColorBrewer::brewer.pal(8, "Set3"), RColorBrewer::brewer.pal(6, "Set2"))[1:length(levels(counts$V1))])
def<- merge(counts, jColors, by="V1")

par(mar=c(10,15,5,10), xpd=T)
barplot(as.numeric(as.character(def$V3)), names.arg = def$cell_line, las=2,
        col=as.character(def$color), ylab="", xlab="")
mtext(text = "Contribution of the top non-Control signature", line=2, cex=1.5)
mtext(text = "Cell Lines", line=6.5, side = 1, cex=1.5)
mtext(text = "Proportion", line=3.5, side = 2, cex=1.5)
legend("topright",legend=sort(unique(def$V1)),bty="n", pch=15,
       col=as.character(unique(def$color)),
       cex=1, pt.cex=1, inset=c(-0.25,0.0),x.intersp = 1,y.intersp = 1)
```
