# Supplementary material for: Timing the initiation of multiple myeloma
Source: Nat Commun. 2020 Apr 21;11:1917. doi: 10.1038/s41467-020-15740-9 (PMC7174344; doi:10.1038/s41467-020-15740-9)
Supplement: Supplementary file 11 — Supplementary Data 8 [file 41467_2020_15740_MOESM11_ESM.html]

Distinct mutational processes as molecular clock in the CoMMpass data set


# Distinct mutational processes as molecular clock in the CoMMpass data set

## Introduction

Normal and tumor somatic cells acquire mutations over the course of their life-time. The final multiple myeloma mutational profile is shaped by 8 main mutational processes. Two of these processes (i.e SBS1 and SBS5) have been reported to introduce mutations throughout life at a constant rate. Consequently, the number of SBS1 and SBS5 mutations in each sample will be proportional to the chronological age. To test the correlation between patients’ age and SBS1/SBS5 mutational burden in multiple myeloma, we investigated the entire CoMMpass data set using both linear regression and linear mixed effect models.

## Libraries

```
packages <- c('readr', 'tidyr', "splitstackshape", "plyr", "dplyr", "ggplot2", "ggpubr", "reshape2", "magrittr", "lme4", "lmerTest", "knitr",
              "ggplot2","reshape2", "MASS", "RColorBrewer", "stringr", "deconstructSigs", "BSgenome.Hsapiens.UCSC.hg19",
              "stringi", "tibble", "pander", "RColorBrewer", "merTools")

invisible(suppressWarnings(suppressMessages(lapply(packages, library, character.only = TRUE))))

# Axis rotate function
rotatedAxisElementText = function(angle,position='x'){
    angle     = angle[1]; 
    position  = position[1]
    positions = list(x=0,y=90,top=180,right=270)
    if(!position %in% names(positions))
        stop(sprintf("'position' must be one of [%s]",paste(names(positions),collapse=", ")),call.=FALSE)
    if(!is.numeric(angle))
        stop("'angle' must be numeric",call.=FALSE)
    rads  = (angle - positions[[ position ]])*pi/180
    hjust = 0.5*(1 - sin(rads))
    vjust = 0.5*(1 + cos(rads))
    element_text(angle=angle,vjust=vjust,hjust=hjust)
}
```

## Load mutational and clinical data

Signature contribution for each sample estimated using the new mutational signature fitting tool named mmsig (https://github.com/evenrus/mmsig).

```
sig<- read.delim("./data/signatures_SNV_COMMPASS_cosine_no_mm1.txt", stringsAsFactors = F)
colnames(sig)<- gsub("Signature.Subs.0","SBS",colnames(sig))
colnames(sig)<- gsub("Signature.Subs.","SBS",colnames(sig))
sig<- sig[,-9]
sig<- sig[order(sig$sampleID),]
sig$sampleID<- as.character(sig$sampleID)
library(stringr)
out <- str_split_fixed((sig$sampleID),'_',5) 
sig$patients<- paste(out[,1], out[,2], sep="_")
head(sig)
```

```
##         sampleID freq    SBS1    SBS2   SBS5   SBS8   SBS9   SBS13 SBS18
## 1 MMRF_1016_1_BM  274 0.12109 0.00000 0.8789 0.0000 0.0000 0.00000     0
## 2 MMRF_1020_3_BM  458 0.04551 0.00000 0.9545 0.0000 0.0000 0.00000     0
## 3 MMRF_1021_1_BM  269 0.03408 0.00000 0.6931 0.1604 0.0000 0.05292     0
## 4 MMRF_1024_2_BM  297 0.15027 0.00000 0.6469 0.0000 0.2028 0.00000     0
## 5 MMRF_1029_1_BM  230 0.08705 0.00000 0.8532 0.0000 0.0000 0.05978     0
## 6 MMRF_1030_1_BM  249 0.10276 0.03071 0.8087 0.0000 0.0000 0.05779     0
##    patients
## 1 MMRF_1016
## 2 MMRF_1020
## 3 MMRF_1021
## 4 MMRF_1024
## 5 MMRF_1029
## 6 MMRF_1030
```

Age and disease status at the time of sampling for all patients in the CoMMpass study was obtained from the Multiple Myeloma Research Foundation online portal (https://research.themmrf.org).

```
clin<- read.delim("./data/clinical_commpass.txt", sep="\t", stringsAsFactors = F)
colnames(clin)[1]<- "sample"
clin2<- clin[,c("sample","DEMOG_PATIENTAGE")]
colnames(clin2)[1]<-"patients"
head(clin2)
```

```
##    patients DEMOG_PATIENTAGE
## 1 MMRF_1007               78
## 2 MMRF_1011               50
## 3 MMRF_1013               66
## 4 MMRF_1014               64
## 5 MMRF_1016               56
## 6 MMRF_1017               75
```

```
time<- read.delim("./data/MMRF_CoMMpass_IA11_Seq_QC_Summary.txt", sep="\t", header=T, stringsAsFactors = F)
time_exo<- time[time$MMRF_Release_Status %in% c("Exome-MutOnly" ,"Exome-Neither", "Exome-All"), ]
time2<- time_exo[,c("Patients..KBase_Patient_ID","Visits..Study.Visit.ID","Visits..Reason_For_Collection","Creation.Date")]
colnames(time2)<-c("patients","sample","status","date")
time2 <- time2 %>%
  group_by(patients) %>%
  mutate(n_samples = length(unique(sample))) %>%
  as.data.frame()
head(time2)
```

```
##    patients      sample   status       date n_samples
## 1 MMRF_1032 MMRF_1032_1 Baseline 03/29/2013         1
## 2 MMRF_1032 MMRF_1032_1 Baseline 04/01/2013         1
## 3 MMRF_1252 MMRF_1252_1 Baseline 04/01/2013         1
## 4 MMRF_1231 MMRF_1231_1 Baseline 04/01/2013         1
## 5 MMRF_1231 MMRF_1231_1 Baseline 04/01/2013         1
## 6 MMRF_1252 MMRF_1252_1 Baseline 04/01/2013         1
```

## Linear Model

Linear regression models were fit to test the association between patients’ age and the number of mutations attributed to each signature: SBS1, SBS2, SBS5, SBS8, SBS9, SBS13, SBS18.

```
sig_lin<- sig[grep("_1_BM",sig$sampleID),]
sig_lin$patients<- gsub("_1_BM","", sig_lin$sampleID)
fin<- merge(sig_lin, clin2, by="patients")
fin<- fin[complete.cases(fin),]

fin[,4:10]<- apply(fin[,4:10], 2, function(x){x * fin$freq})

code<- c(4:10)

for(j in (1:length(code)))
{

test<- cbind.data.frame(fin$DEMOG_PATIENTAGE, fin[,code[j]])
colnames(test)<-c("Age","sig")
p<- ggplot(test, aes(x=Age, y=sig)) +
  geom_point()+
  geom_smooth(method=lm) +
  xlim(20,95) + labs(x = "Age (Years)", y=colnames(fin)[code[j]])+
  theme(
    # plot.title = element_text(color="red", size=14, face="bold.italic"),
    axis.title.x = element_text(size=20),
    axis.title.y = element_text(size=20),
    axis.text.x = element_text(angle = 0, hjust = 1, size=15),
    axis.text.y = element_text(angle = 0, hjust = 1, size=15))
plot(p)
print(summary(lm(as.numeric(as.character(fin[,code[j]]))~fin$DEMOG_PATIENTAGE)))

}
```

```
## 
## Call:
## lm(formula = as.numeric(as.character(fin[, code[j]])) ~ fin$DEMOG_PATIENTAGE)
## 
## Residuals:
##    Min     1Q Median     3Q    Max 
##  -32.8  -13.2   -5.0    7.5  324.6 
## 
## Coefficients:
##                      Estimate Std. Error t value Pr(>|t|)    
## (Intercept)            9.0315     5.3429    1.69  0.09137 .  
## fin$DEMOG_PATIENTAGE   0.2971     0.0823    3.61  0.00032 ***
## ---
## Signif. codes:  0 '***' 0.001 '**' 0.01 '*' 0.05 '.' 0.1 ' ' 1
## 
## Residual standard error: 24.5 on 762 degrees of freedom
## Multiple R-squared:  0.0168, Adjusted R-squared:  0.0155 
## F-statistic:   13 on 1 and 762 DF,  p-value: 0.000325
```

```
## 
## Call:
## lm(formula = as.numeric(as.character(fin[, code[j]])) ~ fin$DEMOG_PATIENTAGE)
## 
## Residuals:
##    Min     1Q Median     3Q    Max 
##    -56    -47    -44    -35   6490 
## 
## Coefficients:
##                      Estimate Std. Error t value Pr(>|t|)
## (Intercept)            63.688     69.182    0.92     0.36
## fin$DEMOG_PATIENTAGE   -0.279      1.065   -0.26     0.79
## 
## Residual standard error: 318 on 762 degrees of freedom
## Multiple R-squared:  8.99e-05,   Adjusted R-squared:  -0.00122 
## F-statistic: 0.0685 on 1 and 762 DF,  p-value: 0.794
```

```
## 
## Call:
## lm(formula = as.numeric(as.character(fin[, code[j]])) ~ fin$DEMOG_PATIENTAGE)
## 
## Residuals:
##    Min     1Q Median     3Q    Max 
## -217.5  -61.0   -2.9   49.9  569.5 
## 
## Coefficients:
##                      Estimate Std. Error t value     Pr(>|t|)    
## (Intercept)           123.511     19.348    6.38 0.0000000003 ***
## fin$DEMOG_PATIENTAGE    1.556      0.298    5.22 0.0000002270 ***
## ---
## Signif. codes:  0 '***' 0.001 '**' 0.01 '*' 0.05 '.' 0.1 ' ' 1
## 
## Residual standard error: 88.8 on 762 degrees of freedom
## Multiple R-squared:  0.0346, Adjusted R-squared:  0.0333 
## F-statistic: 27.3 on 1 and 762 DF,  p-value: 0.000000227
```

```
## 
## Call:
## lm(formula = as.numeric(as.character(fin[, code[j]])) ~ fin$DEMOG_PATIENTAGE)
## 
## Residuals:
##    Min     1Q Median     3Q    Max 
## -13.32 -10.36  -9.50  -7.77 161.95 
## 
## Coefficients:
##                      Estimate Std. Error t value Pr(>|t|)
## (Intercept)            1.8527     5.0446    0.37     0.71
## fin$DEMOG_PATIENTAGE   0.1233     0.0777    1.59     0.11
## 
## Residual standard error: 23.2 on 762 degrees of freedom
## Multiple R-squared:  0.0033, Adjusted R-squared:  0.00199 
## F-statistic: 2.52 on 1 and 762 DF,  p-value: 0.113
```

```
## 
## Call:
## lm(formula = as.numeric(as.character(fin[, code[j]])) ~ fin$DEMOG_PATIENTAGE)
## 
## Residuals:
##    Min     1Q Median     3Q    Max 
##  -23.1  -20.8  -20.1   19.7  264.0 
## 
## Coefficients:
##                      Estimate Std. Error t value Pr(>|t|)   
## (Intercept)            25.381      8.157    3.11   0.0019 **
## fin$DEMOG_PATIENTAGE   -0.075      0.126   -0.60   0.5509   
## ---
## Signif. codes:  0 '***' 0.001 '**' 0.01 '*' 0.05 '.' 0.1 ' ' 1
## 
## Residual standard error: 37.4 on 762 degrees of freedom
## Multiple R-squared:  0.000467,   Adjusted R-squared:  -0.000845 
## F-statistic: 0.356 on 1 and 762 DF,  p-value: 0.551
```

```
## 
## Call:
## lm(formula = as.numeric(as.character(fin[, code[j]])) ~ fin$DEMOG_PATIENTAGE)
## 
## Residuals:
##    Min     1Q Median     3Q    Max 
##  -36.8  -26.8  -24.6  -20.1 2726.1 
## 
## Coefficients:
##                      Estimate Std. Error t value Pr(>|t|)
## (Intercept)            44.933     34.818    1.29     0.20
## fin$DEMOG_PATIENTAGE   -0.303      0.536   -0.57     0.57
## 
## Residual standard error: 160 on 762 degrees of freedom
## Multiple R-squared:  0.000419,   Adjusted R-squared:  -0.000893 
## F-statistic: 0.319 on 1 and 762 DF,  p-value: 0.572
```

```
## 
## Call:
## lm(formula = as.numeric(as.character(fin[, code[j]])) ~ fin$DEMOG_PATIENTAGE)
## 
## Residuals:
##    Min     1Q Median     3Q    Max 
##  -2.97  -1.98  -1.68  -1.34  67.12 
## 
## Coefficients:
##                      Estimate Std. Error t value Pr(>|t|)
## (Intercept)           -1.0168     1.7189   -0.59     0.55
## fin$DEMOG_PATIENTAGE   0.0428     0.0265    1.62     0.11
## 
## Residual standard error: 7.89 on 762 degrees of freedom
## Multiple R-squared:  0.00343,    Adjusted R-squared:  0.00212 
## F-statistic: 2.62 on 1 and 762 DF,  p-value: 0.106
```

## Linear Mixed Effects model selection to estimate patient-specific mutation rates

Next, we applied linear mixed effect models to confirm the clock-like nature of SBS5 and SBS1 in multiple myeloma, similar to recent reports from tumor and normal tissues (Mitchell et al, Cell 2018, June-Koo et al, Cell 2019, Moore et al, Biorxiv 2018 - https://doi.org/10.1101/505685, Lee-Six et al, Nature 2019). Linear mixed effect model allows estimation of patient-specific mutation rates in patients with sequential samples. Patients with samples obtained at diagnosis and one or more relapse were included.

### SBS5 mutation rate

First, we determined the optimal parameters for linear mixed effects modeling. Below, we compare three ways of modeling the relationship between age and SBS5 mutation counts: 1) including patient-specific and population intercept; 2) including patient specific intercept only and 3) constraining the intercept to 0.

```
## Combine signature and clinical data to select patients with samples collected at diagnosis and at relapse

sig$sample<- gsub("_BM", "", sig$sampleID)
sig3<- sig[sig$patients%in% names(table(sig$patients))[which(table(sig$patients)>1)], ]

int<- merge(sig3, time2,by=c("patients","sample"))
clin3<- clin[,c(1,3,13,14)]
colnames(clin3)[1]<-"patients"
df<- merge(int, clin3, by=c("patients"))
df<- unique(df)
df<- df[order(df$sample),]
df2<- df[df$patients%in% names(table(df$patients))[which(table(df$patients)>1)], ]

sampleID<- unique((df2$patients))
all_df<- list()
for(i in (1:length(sampleID)))
{
  


  df_sam<- df[df$patients ==sampleID[i],]
  df_sam$age<- df_sam$DEMOG_PATIENTAGE + (as.numeric(as.Date(as.character(df_sam$date), format="%m/%d/%Y") - as.Date(as.character(df_sam$date)[1],   format="%m/%d/%Y")))/360
 all_df[[i]]<- df_sam
}
all_df2<- do.call("rbind", all_df)
all_df2_prog_list<- all_df2[all_df2$status =="Confirm Progression",]
all_df2_prog2<- all_df2[all_df2$patients %in% all_df2_prog_list$patients,]
all_df2_prog <- all_df2_prog2[all_df2_prog2$status %in% c("Baseline","Confirm Progression"),]
all_df2_prog <- all_df2_prog[all_df2_prog$n_samples > 1,]
```

```
## lmer with and without variable slope and with and without origin constrained for SBS5

all_df2_prog$burden<- all_df2_prog$SBS5 * all_df2_prog$freq

### free intercepts
muts.per.year.lmer.with.pt.intercepts <-lmer(burden ~ age + (1 + age | patients), data=all_df2_prog, REML=FALSE)
```

```
## Warning in checkConv(attr(opt, "derivs"), opt$par, ctrl = control$checkConv, :
## unable to evaluate scaled gradient
```

```
## Warning in checkConv(attr(opt, "derivs"), opt$par, ctrl = control$checkConv, :
## Model failed to converge: degenerate Hessian with 1 negative eigenvalues
```

```
## Warning: Model failed to converge with 1 negative eigenvalue: -5.7e-01
```

```
### patient-specific intercept constrained to 0 
muts.per.year.lmer.with.pop.intercept <- lmer(burden ~ age + (0 + age | patients), data=all_df2_prog, REML=FALSE)

### population and patient-specific intercepts constrained to 0
muts.per.year.lmer <- lmer(burden ~ age + 0 + (age + 0 | patients ), data=all_df2_prog, REML=FALSE)


### print the results

print(summary(muts.per.year.lmer))
```

```
## Linear mixed model fit by maximum likelihood . t-tests use Satterthwaite's
##   method [lmerModLmerTest]
## Formula: burden ~ age + 0 + (age + 0 | patients)
##    Data: all_df2_prog
## 
##      AIC      BIC   logLik deviance df.resid 
##   1827.7   1836.8   -910.9   1821.7      151 
## 
## Scaled residuals: 
##    Min     1Q Median     3Q    Max 
## -1.740 -0.485 -0.135  0.472  2.083 
## 
## Random effects:
##  Groups   Name Variance Std.Dev.
##  patients age     1.68   1.3    
##  Residual      3764.71  61.4    
## Number of obs: 154, groups:  patients, 72
## 
## Fixed effects:
##     Estimate Std. Error     df t value            Pr(>|t|)    
## age    3.767      0.172 69.868    21.9 <0.0000000000000002 ***
## ---
## Signif. codes:  0 '***' 0.001 '**' 0.01 '*' 0.05 '.' 0.1 ' ' 1
```

```
print(summary(muts.per.year.lmer.with.pt.intercepts))
```

```
## Linear mixed model fit by maximum likelihood . t-tests use Satterthwaite's
##   method [lmerModLmerTest]
## Formula: burden ~ age + (1 + age | patients)
##    Data: all_df2_prog
## 
##      AIC      BIC   logLik deviance df.resid 
##   1833.1   1851.4   -910.6   1821.1      148 
## 
## Scaled residuals: 
##    Min     1Q Median     3Q    Max 
## -1.733 -0.463 -0.152  0.466  2.108 
## 
## Random effects:
##  Groups   Name        Variance Std.Dev. Corr
##  patients (Intercept) 1115.845 33.404       
##           age            0.873  0.935   0.56
##  Residual             3760.702 61.325       
## Number of obs: 154, groups:  patients, 72
## 
## Fixed effects:
##             Estimate Std. Error     df t value Pr(>|t|)    
## (Intercept)   22.063     55.230 38.140    0.40  0.69177    
## age            3.426      0.845 55.016    4.05  0.00016 ***
## ---
## Signif. codes:  0 '***' 0.001 '**' 0.01 '*' 0.05 '.' 0.1 ' ' 1
## 
## Correlation of Fixed Effects:
##     (Intr)
## age -0.979
## convergence code: 0
## unable to evaluate scaled gradient
## Model failed to converge: degenerate  Hessian with 1 negative eigenvalues
```

```
print(summary(muts.per.year.lmer.with.pop.intercept))
```

```
## Linear mixed model fit by maximum likelihood . t-tests use Satterthwaite's
##   method [lmerModLmerTest]
## Formula: burden ~ age + (0 + age | patients)
##    Data: all_df2_prog
## 
##      AIC      BIC   logLik deviance df.resid 
##   1829.6   1841.8   -910.8   1821.6      150 
## 
## Scaled residuals: 
##    Min     1Q Median     3Q    Max 
## -1.732 -0.501 -0.135  0.470  2.088 
## 
## Random effects:
##  Groups   Name Variance Std.Dev.
##  patients age     1.67   1.29   
##  Residual      3779.31  61.48   
## Number of obs: 154, groups:  patients, 72
## 
## Fixed effects:
##             Estimate Std. Error      df t value Pr(>|t|)    
## (Intercept)   17.092     51.325 106.803    0.33     0.74    
## age            3.506      0.802  97.649    4.37 0.000031 ***
## ---
## Signif. codes:  0 '***' 0.001 '**' 0.01 '*' 0.05 '.' 0.1 ' ' 1
## 
## Correlation of Fixed Effects:
##     (Intr)
## age -0.977
```

```
### ANOVA test  
pandoc.table(anova(muts.per.year.lmer, muts.per.year.lmer.with.pop.intercept, muts.per.year.lmer.with.pt.intercepts), split.tables  = "Inf")
```

```
## 
## -----------------------------------------------------------------------------------------------------------------
##                   &nbsp;                     Df   AIC    BIC    logLik   deviance   Chisq    Chi Df   Pr(>Chisq) 
## ------------------------------------------- ---- ------ ------ -------- ---------- -------- -------- ------------
##           **muts.per.year.lmer**             3    1828   1837   -910.9     1822       NA       NA         NA     
## 
##  **muts.per.year.lmer.with.pop.intercept**   4    1830   1842   -910.8     1822     0.1091     1        0.7412   
## 
##  **muts.per.year.lmer.with.pt.intercepts**   6    1833   1851   -910.6     1821     0.4707     2        0.7903   
## -----------------------------------------------------------------------------------------------------------------
```

There was no difference in the model fit with and without intercept. Therefore, we went forward with the most parsimonious model, i.e. intercept constrained to zero.

Below we show the observed SBS5 mutation and age data for each patient, along with patient-specific slopes (colored lines) and the average slope across the cohort (dashed black line).

```
## Generate SBS5 mutation rate per year estimate for each patient

allsamples.df <- all_df2_prog

muts.per.year.lmer <- lmer(burden ~ age + 0 + (age + 0 | patients ), data=allsamples.df, REML=FALSE)


allsamples.df$Pt.fitted.num.mutrate <- (fixef(muts.per.year.lmer)["age"] +                                                   ranef(muts.per.year.lmer)$patients[allsamples.df$patients,"age"]) * allsamples.df$age


ggplot(data = allsamples.df, aes(x=age, y=burden, color=patients)) + 
  geom_segment(aes(x=0, y = 0, xend=age, yend=Pt.fitted.num.mutrate, colour=patients), data=allsamples.df) +
  geom_abline(intercept=0,slope=fixef(muts.per.year.lmer), size=3) +
  geom_point(shape=16) +
  xlim(c(0,100)) + ylim(c(0,700)) + theme(legend.position="none")+ 
 labs(x = "Age (Years)", y="SBS5")+ 
  theme(
    # plot.title = element_text(color="red", size=14, face="bold.italic"),
    axis.title.x = element_text(size=20),
    axis.title.y = element_text(size=20), 
    axis.text.x = element_text(angle = 0, hjust = 1, size=15),
    axis.text.y = element_text(angle = 0, hjust = 1, size=15)
  )
```

Below, we show the estimated SBS5 mutation rate with standard deviation for each patient, as determined by the LME model. The average SBS5 mutation rate is shown as a horizontal red line. Dots with error-bars show the patient-specific mutation rates.

```
# Variable setup

mutrate_patients <- allsamples.df %>%
  distinct(patients)

# SIMPLE MODEL

# obtaining the population estimate for mutation rate
mutrate_patients$fixed_effect <- fixef(muts.per.year.lmer)

# simulating the uncertainty in patient-specific adjustment factor for mutation rate (random effect)
random_effect_sim <- REsim(muts.per.year.lmer, n.sims = 1000)
random_effect_sim <- random_effect_sim[c(2,4,6)]
names(random_effect_sim) <- c("patients", "ranef_mean", "ranef_sd")

mutrate_patients <- mutrate_patients %>%
    left_join(random_effect_sim, by = "patients") %>%
    mutate(sd_upper = fixed_effect + ranef_mean + ranef_sd,
           pt_est = fixed_effect + ranef_mean,
           sd_lower = fixed_effect + ranef_mean - ranef_sd) %>%
    arrange(pt_est)
```

```
ggplot(data = mutrate_patients)+
    geom_point(aes(reorder(patients, pt_est), pt_est), size = 3)+
    geom_errorbar(aes(x = reorder(patients, pt_est), ymin = sd_lower, ymax = sd_upper))+
    geom_hline(yintercept = fixef(muts.per.year.lmer), size = 1, col = "darkred")+
    scale_color_brewer(palette = "Set1")+
    scale_y_continuous(limits = c(0,10))+
    labs(y = "SBS5 mutation rate",
         col = "Number of samples available")+
    theme(axis.title.x = element_blank(),
          axis.text.x = rotatedAxisElementText(90, "top"),
          axis.text = element_text(size = 10),
          legend.position = "top")
```

```
ggsave("SBS5_mutation_rate_commpass.pdf", height = 3, width = 10)
```

Finally, we show the average SBS5 mutation rate across the cohort (exome-wide), along with the between-patient standard deviation.

```
# cohort average rate
fixef_est <- as.numeric(fixef(muts.per.year.lmer))

std_err_fixef <- summary(muts.per.year.lmer)$coefficients[1,2]

# 95 % CI for the cohort average
upperCI <-  fixef_est + 1.96*std_err_fixef
lowerCI <-  fixef_est  - 1.96*std_err_fixef

# standard deviation of between-patient differences
between_pts_sd <- sd(mutrate_patients$pt_est)

# Between-patient standard deviation as percent of the average SBS5 mutation rate
between_pts_percent <- between_pts_sd/fixef_est*100

paste0("Average SBS5 mutation rate: ", round(fixef_est,2), " (", round(lowerCI, 2), "-", round(upperCI, 2), ")")
```

```
## [1] "Average SBS5 mutation rate: 3.77 (3.43-4.1)"
```

```
paste0("Between-patient standard deviation: ", round(between_pts_sd, 2), " (", round(between_pts_percent, 2), " %)")
```

```
## [1] "Between-patient standard deviation: 1.16 (30.72 %)"
```

### SBS1 mutation rate

SBS1 was the only mutational process other than SBS5 that showed a linear association with age, consistent with a clock-like nature.

Below we show the observed SBS1 mutation and age data for each patient, along with patient-specific slopes (colored lines) and the average slope across the cohort (dashed black line). As for SBS5, we constrained the intercept to zero in the linear mixed effects model.

```
all_df2_prog$burden<- all_df2_prog$SBS1 * all_df2_prog$freq

### free intercepts
muts.per.year.lmer.with.pt.intercepts <-lmer(burden ~ age + (1 + age | patients), data=all_df2_prog, REML=FALSE)
```

```
## boundary (singular) fit: see ?isSingular
```

```
## Warning: Model failed to converge with 1 negative eigenvalue: -1.0e+02
```

```
### patient-specific intercept constrained to 0 
muts.per.year.lmer.with.pop.intercept <- lmer(burden ~ age + (0 + age | patients), data=all_df2_prog, REML=FALSE)

### population and patient-specific intercepts constrained to 0
muts.per.year.lmer <- lmer(burden ~ age + 0 + (age + 0 | patients ), data=all_df2_prog, REML=FALSE)

### print the results

print(summary(muts.per.year.lmer))
```

```
## Linear mixed model fit by maximum likelihood . t-tests use Satterthwaite's
##   method [lmerModLmerTest]
## Formula: burden ~ age + 0 + (age + 0 | patients)
##    Data: all_df2_prog
## 
##      AIC      BIC   logLik deviance df.resid 
##   1261.0   1270.2   -627.5   1255.0      151 
## 
## Scaled residuals: 
##    Min     1Q Median     3Q    Max 
## -3.151 -0.318 -0.069  0.248  4.666 
## 
## Random effects:
##  Groups   Name Variance Std.Dev.
##  patients age   0.0866  0.294   
##  Residual      58.5498  7.652   
## Number of obs: 154, groups:  patients, 72
## 
## Fixed effects:
##     Estimate Std. Error      df t value            Pr(>|t|)    
## age   0.4158     0.0361 72.5801    11.5 <0.0000000000000002 ***
## ---
## Signif. codes:  0 '***' 0.001 '**' 0.01 '*' 0.05 '.' 0.1 ' ' 1
```

```
print(summary(muts.per.year.lmer.with.pt.intercepts))
```

```
## Linear mixed model fit by maximum likelihood . t-tests use Satterthwaite's
##   method [lmerModLmerTest]
## Formula: burden ~ age + (1 + age | patients)
##    Data: all_df2_prog
## 
##      AIC      BIC   logLik deviance df.resid 
##   1266.6   1284.8   -627.3   1254.6      148 
## 
## Scaled residuals: 
##    Min     1Q Median     3Q    Max 
## -3.109 -0.291 -0.076  0.244  4.707 
## 
## Random effects:
##  Groups   Name        Variance Std.Dev. Corr
##  patients (Intercept)  0.0000  0.000        
##           age          0.0856  0.293     NaN
##  Residual             58.8008  7.668        
## Number of obs: 154, groups:  patients, 72
## 
## Fixed effects:
##             Estimate Std. Error     df t value Pr(>|t|)   
## (Intercept)   -6.496      9.837 93.542   -0.66   0.5107   
## age            0.516      0.157 89.604    3.30   0.0014 **
## ---
## Signif. codes:  0 '***' 0.001 '**' 0.01 '*' 0.05 '.' 0.1 ' ' 1
## 
## Correlation of Fixed Effects:
##     (Intr)
## age -0.973
## convergence code: 0
## boundary (singular) fit: see ?isSingular
```

```
print(summary(muts.per.year.lmer.with.pop.intercept))
```

```
## Linear mixed model fit by maximum likelihood . t-tests use Satterthwaite's
##   method [lmerModLmerTest]
## Formula: burden ~ age + (0 + age | patients)
##    Data: all_df2_prog
## 
##      AIC      BIC   logLik deviance df.resid 
##   1262.6   1274.8   -627.3   1254.6      150 
## 
## Scaled residuals: 
##    Min     1Q Median     3Q    Max 
## -3.110 -0.291 -0.076  0.245  4.707 
## 
## Random effects:
##  Groups   Name Variance Std.Dev.
##  patients age   0.0856  0.293   
##  Residual      58.7804  7.667   
## Number of obs: 154, groups:  patients, 72
## 
## Fixed effects:
##             Estimate Std. Error     df t value Pr(>|t|)   
## (Intercept)   -6.493      9.839 95.056   -0.66   0.5109   
## age            0.516      0.157 90.395    3.30   0.0014 **
## ---
## Signif. codes:  0 '***' 0.001 '**' 0.01 '*' 0.05 '.' 0.1 ' ' 1
## 
## Correlation of Fixed Effects:
##     (Intr)
## age -0.973
```

```
### ANOVA test  
kable(anova(muts.per.year.lmer, muts.per.year.lmer.with.pop.intercept, muts.per.year.lmer.with.pt.intercepts))
```

|  | Df | AIC | BIC | logLik | deviance | Chisq | Chi Df | Pr(>Chisq) |
| --- | --- | --- | --- | --- | --- | --- | --- | --- |
| muts.per.year.lmer | 3 | 1261 | 1270 | -627.5 | 1255 | NA | NA | NA |
| muts.per.year.lmer.with.pop.intercept | 4 | 1263 | 1275 | -627.3 | 1255 | 0.4309 | 1 | 0.5115 |
| muts.per.year.lmer.with.pt.intercepts | 6 | 1267 | 1285 | -627.3 | 1255 | 0.0000 | 2 | 1.0000 |

```
allsamples.df <- all_df2_prog
muts.per.year.lmer <- lmer(burden ~ age + 0 + (age + 0 | patients ), data=allsamples.df, REML=FALSE)

allsamples.df$Pt.fitted.num.mutrate <- (fixef(muts.per.year.lmer)["age"] +                                                   ranef(muts.per.year.lmer)$patients[allsamples.df$patients,"age"]) * allsamples.df$age


ggplot(data = allsamples.df, aes(x=age, y=burden, color=patients)) + 
  geom_segment(aes(x=0, y = 0, xend=age, yend=Pt.fitted.num.mutrate, colour=patients), data=allsamples.df) +
  geom_abline(intercept=0,slope=fixef(muts.per.year.lmer), size=3) +
  geom_point(shape=16) +
  xlim(c(0,100)) + ylim(c(0,200)) + theme(legend.position="none")+ 
 labs(x = "Age (Years)", y="SBS1")+ 
  theme(
    # plot.title = element_text(color="red", size=14, face="bold.italic"),
    axis.title.x = element_text(size=20),
    axis.title.y = element_text(size=20), 
    axis.text.x = element_text(angle = 0, hjust = 1, size=15),
    axis.text.y = element_text(angle = 0, hjust = 1, size=15)
  )
```
