# Supplementary material for: Timing the initiation of multiple myeloma
Source: Nat Commun. 2020 Apr 21;11:1917. doi: 10.1038/s41467-020-15740-9 (PMC7174344; doi:10.1038/s41467-020-15740-9)
Supplement: Supplementary file 12 — Supplementary Data 9 [file 41467_2020_15740_MOESM12_ESM.html]

Timing the first multi-gain events in Multiple Myeloma


# Timing the first multi-gain events in Multiple Myeloma

## Introduction: Molecular and absolute time in Multiple Myeloma

Tumors contain a record of all mutations acquired in different phases of its life history, which can be re-constructed from whole genome sequencing (WGS) data into a phylogenetic tree. The trunk contains all mutations acquired from the fertilized egg through transformative events up to and including the most recent common ancestor (MRCA). From there the evolutionary trajectory diverges, detectable by whole genome sequencing as one or more distinct branches or subclones.

Timing when landmark events occurred along the phylogenetic tree can be understood in terms of molecular and chronological time.

Molecular time refers to the relative order of events along the cancer phylogenetic tree, estimated here by the corrected ratio of duplicated and non-duplicated mutations within large clonal chromosomal gains. Using a molecular time approach, we previously showed that most large chromosomal gains in multiple myeloma occur within a single early time-window, sometimes followed by additional gains in later distinct time-windows throughout evolutionary time (Maura F et al, Nat Comm 2019a).

Translating from molecular to chronological time requires the use of a molecular clock: a mutational process with constant rate over time. This allows inference to be made about how long it may have taken to acquire a given number of mutations. We have previously published that total mutation burden can be used as a molecular clock in kidney cancer (Mitchell et al, Cell 2018), allowing estimation of the patient age when landmark events occurred in the life history of patients. The same apporach was recently applied to lung cancer (Lee et al, Cell 2019). The present work draws on principles and methodology from the above-mentioned papers.

In multiple myeloma, we found the mutational signature landscape to be more complex than in kidney cancer. As discussed further in the main manuscript and Data S2, SBS5 appears to fulfill the requirements for a molecular clock in multiple myeloma. The SBS5 mutational burden at diagnosis correlates linearly with patient age; and importantly, a patient-specific SBS5 mutation rate can be estimated from sequential sample data using linear mixed models (LME). This is consistent with findings in other cancers and normal cells (Alexandrov et al, Nat Gen 2015, Blokzijl F et al, Nature 2016, Mitchell et al, Cell 2018, Lee et al Cell 2019, Lee-Six et al, Nature 2019, Moore et al, bioRxiv 2018 - https://doi.org/10.1101/505685, ).

We expect SBS5 to behave like a molecular clock in the majority of multiple myeloma tumors; however, there are some hypermutated tumors where this assumption may not hold. Although hypermutator phenotypes in multiple myeloma are largely APOBEC-driven, we cannot exclude involvement of SBS5 as well. Two tumors in our dataset had > 10 000 SNVs and were excluded from all the analyses below. An additional sample (PD26414) was excluded considering its ploidy>4 due to two independent whole genome duplications (Maura et al., 2019a).

In summary, the following analysis consists of three principal parts: 1) confirm the clock-like nature of SBS5 in tumor phylogenies reconstructed from whole genome sequencing data and determine the optimal modeling parameters; 2) estimate the SBS5 mutation rate (mutations/year) for each multiple myeloma patient; and 3) apply this mutation rate to SBS5 mutations acquired before landmark events in tumor evolution to estimate the time elapsed (i.e. patient age) when each event occurred.

## Libraries

```
packages <- c('readr', 'tidyr', "splitstackshape", "plyr", "dplyr", "ggplot2", "ggpubr", "reshape2", "magrittr", "lme4", "lmerTest", "knitr",
              "ggplot2","reshape2", "MASS", "RColorBrewer", "stringr", "deconstructSigs", "BSgenome.Hsapiens.UCSC.hg19",
              "stringi", "tibble", "pander", "RColorBrewer", "merTools")

invisible(suppressWarnings(suppressMessages(lapply(packages, library, character.only = TRUE))))
```

## Load Functions

```
### Molecular time function, from the mol_time R package (Maura et al. 2018)
# 1 extra gain
iter = 1000
triploid.2.1.est <- function(ploidy.1, ploidy.2, bootstrap = TRUE, iter=1000) {
    retv <- list()
    point.est <- ploidy.2 / (ploidy.2 + (ploidy.1 - ploidy.2)/3)
    if (bootstrap) {
        total.muts <- ploidy.1 + ploidy.2
        prob.2 <- ploidy.2 / total.muts
        resamps <- rbinom(n=iter, size=total.muts, p=prob.2)
        ests.bs <- resamps / (resamps + (total.muts - 2*resamps)/3)
        ests.95 <- quantile(ests.bs, c(0.025,0.975))
        # return(c(point.est, ests.95))
        retv$pests <- c(point.est,ests.95)
        retv$noe   <- 1
        retv$boot1 <- ests.bs
    }
    else {retv$pests <- point.est} # return(point.est)}
    
    return(retv)
}

### Helper functions
# Helper functions
color<- c(RColorBrewer::brewer.pal(12, "Paired"), RColorBrewer::brewer.pal(12, "Set3"), RColorBrewer::brewer.pal(8, "Dark2"), 
          RColorBrewer::brewer.pal(8, "Accent"), RColorBrewer::brewer.pal(9, "Spectral"))

# Axis rotate function
rotatedAxisElementText = function(angle,position='x'){
    angle     = angle[1]; 
    position  = position[1]
    positions = list(x=0,y=90,top=180,right=270)
    if(!position %in% names(positions))
        stop(sprintf("'position' must be one of [%s]",paste(names(positions),collapse=", ")),call.=FALSE)
    if(!is.numeric(angle))
        stop("'angle' must be numeric",call.=FALSE)
    rads  = (angle - positions[[ position ]])*pi/180
    hjust = 0.5*(1 - sin(rads))
    vjust = 0.5*(1 + cos(rads))
    element_text(angle=angle,vjust=vjust,hjust=hjust)
}

### plot linear regression with ggplot

ggplotRegression <- function(fit, colVar, code){
    
    ggplot(fit$model, aes_string(x = names(fit$model)[2], y = names(fit$model)[1])) + 
        geom_point(aes(color=colVar), size = 3) +
        guides(col = guide_legend(ncol = 2))+
        labs(x = code) +
        guides(col = F)+
        theme(text = element_text(size=12),
              axis.title.y = element_blank())+
        stat_smooth(method = "lm", col = "red") +
        labs(title = paste("Adj R2 = ",signif(summary(fit)$adj.r.squared, 5), 
                           " P =",signif(summary(fit)$coef[2,4], 5)))
}

### mmsig -- as available on github.com/evenrus/mmsig
suppressWarnings(suppressMessages(source("util/mmsig.R")))
```

## Load data

```
### Loding mutational signature data for each cluster of mutations identified by the Dirichlet Process (DP) in each patient

sig_DP<- read.delim("data/all_patients_DP_contribution.txt", sep="\t", stringsAsFactors = F, row.names = 1) 
sig_DP$sampleID<- rownames(sig_DP)

#### data frame with signature 5 contribution for each cluster
sig_2_DP<- cbind.data.frame(sig_DP$sampleID, sig_DP$Signature.Subs.05  * sig_DP$mutations) 
colnames(sig_2_DP)<-c("branches","burden")

### Loading phylogenetic tree structure

tree<- read.delim("data/summary_fro_timing.txt", sep="\t", header=T, stringsAsFactors = F) 

#### file with phylogenetic tree structure reconstructed based on DP clusters using the pigeonhole principle
#### V0D58I had one sample and two clusters with  >90%. These violate the pigeonhole principle. We collapsed the clusters together, considering the resulting cluster as the trunk.

tree$tree_structure[tree$patient == "V0D58I"]<-"2>1"
sig_2_DP$burden[sig_2_DP$branches == "V0D58I_2"]<- 1447.15 + 693.27
sig_2_DP<- sig_2_DP[-which(sig_2_DP$branches == "V0D58I_3"),]
out <- do.call("rbind",strsplit(tree$tree_structure,"") )
out_df<- as.data.frame(out)
out_df[,1:4]<- apply(out_df[,1:4], 2, function(x) paste(tree$patient, x, sep="_"))
tree$clonal_five<- NA
tree$burden<- NA
for(i in (1:nrow(tree)))
{
 vec<-  as.data.frame(strsplit(tree$tree_structure[i],">"))
 vec2<- as.numeric(as.character(vec[,1]))
 names_branch<- paste(tree$patient[i], vec2, sep="_")
 sig_bran<- sig_2_DP[which(sig_2_DP$branches %in% names_branch),] #### file sig_2 with signature 5 contribution for each cluster
 tree$burden[i]<-  sum(sig_bran$burden)
 tree$clonal_five[i] <- sig_bran[sig_bran$branches == names_branch[1],2]
}

### Cancer Cell Fractions

clin<- read.delim("data/Supplementary_Table_1.txt", sep="\t")
colnames(clin)[1]<-"sample_ID"
def<- merge(tree, clin[,c(1:3,5,9,10)], by="sample_ID")
def <- def %>%
    mutate(Stage = factor(ifelse(Stage %in% c("MGUS", "SMM"), "Smoldering MM",
                            ifelse(Stage == "MM DG", "Newly diagnosed MM", "Relapsed MM")),
                          levels = c("Smoldering MM", "Newly diagnosed MM", "Relapsed MM")))

def2<- def[,c("sample_ID","patient","patient","tree_structure","Num.clonal","Num.branch.mutations",
              "burden","clonal_five","Age","code_btranch", "CCF",  "coverage", "Stage", "Ploidy")]
colnames(def2)<- c("Paz","Sample","LRISample","Name","Num.clonal.all","Num.branch.mutations",
                   "Num.mutations","Num.clonal" ,"Age","code_btranch", "CCF", "Coverage", "Stage", "Ploidy")

### Remove hypermutated patients, here defined by samples with more than 10.000 SNVs

def2 <- def2[!def2$Sample %in% c("MMRC006BBM","V0D57H", "PD26414"),]

### Create two data frames, one with all patients and one with only patients with more than one evolutionary trajectory, i.e. branching evolution

branch.df3 <- def2[def2$code_btranch =="branch",] #### only multiple evolutionary trajectories, i.e. branching evolution
branch.df2<- def2 #### all patients

# Import signature reference
sig_ref <- read.delim("data/mm_signature_definitions.txt", stringsAsFactors = F, header=T) 

# file with all mol_time info
mut_cnv_order <-  read.csv("data/mol_time_mut_order_cnv.csv", stringsAsFactors = F) 

# mutational signatures active in each sample
load("data/sample.sigt.profs.Rdata") # sample.sigt.profs
```

## Exploratory analysis of SBS5 mutation burden in branches

To estimate the patient-specific SBS5 mutation rate per gigabase (Gb) year, we applied linear mixed effect models (LME) to phylogenetic branches. To add up the SBS5 mutations in each branch, we drew a line from the tip of each branch, via any larger branches and down through the trunk, following the pigeonhole principle as for the tree reconstruction (Nik-Zainal, Cell 2012). In this way, patients with branching evolution contribute more than one observation to the analysis (n = number of terminal branches), allowing more accurate estimation of the mutation rate. We also avoid overestimating the mutation rate by pooling together mutations that were acquired in parallel (in distinct cell populations).

For example, patient PD26403 (Supplementary Figure 9) had four clusters of mutations. The trunk (cluster 1) gave rise directly to three branches (clusters 2-4), one very short and two longer. After the MRCA (end of the trunk), mutations were acquired independently in each of the three branches. This patient contributed three phylogenies to the mutation rate estimation, consisting of the following clusters of mutations added together: 1+2, 1+3 and 1+4.

Before going on to statistical modeling, we explored the relationship between SBS5 mutation burden, age, and other factors which may affect the observed mutational burden.

Below we show the SBS5 mutations for each phylogenetic branch (y-axis) as it relates to other age and other factors which may affect mutation burden identified by WGS (x-axis), colored by patients (each patient may contribute one or more points/branches).

```
expl_1 <-ggplotRegression(lm(Num.mutations ~ Age, data = branch.df2), branch.df2$LRISample, "Age (years)")
expl_2 <-ggplotRegression(lm(Num.mutations ~ CCF, data = branch.df2), branch.df2$LRISample, "Tumor purity")
expl_3 <-ggplotRegression(lm(Num.mutations ~ Coverage, data = branch.df2), branch.df2$LRISample, "Average sequencing coverage")
expl_4 <-ggplotRegression(lm(Num.mutations ~ Ploidy, data = branch.df2), branch.df2$LRISample, "Ploidy")


expl_comb <- ggarrange(expl_1, expl_2, expl_3, expl_4, nrow = 2, ncol = 2)
expl_comb <- annotate_figure(expl_comb, left = "SBS5 mutations in phylogenetic branch")

expl_comb
```

As shown above, SBS5 mutational burden was strongly correlated with patient age. There was a non-significant trend for higher mutational burden in samples with higher purity, as one might expect for technical reasons. Ploidy and sequencing coverage was not related to SBS5 mutation burden.

Below we show the relationship between age and SBS5 mutation burden, colored by disease stage at the time of sampling, i.e. smoldering multiple myeloma, newly diagnosed multiple myeloma or relapsed disease. Fitted linear regression lines for each disease stage showed widely overlapping standard errors (shaded areas).

```
ggplot(filter(branch.df2, !is.na(Stage)), aes(Age, Num.mutations, col = Stage)) + 
        geom_point(size = 3) +
        guides(col = guide_legend(ncol = 2))+
        scale_color_brewer(palette = "Set1")+
        labs(x = "Age (years)",
             y = "SBS5 mutations in phylogenetic branch",
             col = "Disease stage at sampling") +
        theme(text = element_text(size=12))+
        stat_smooth(method = "lm")
```

## Linear Mixed Effects model selection to estimate patient-specific mutation rates

### Model selection: Constrained versus unconstrained intercepts

The LME framework allows estimation of patient-specific slopes and intercepts (“random effects”) as well as population average values (“fixed effects”, similar to standard linear regression). For each effect (fixed or random), we can specify whether to estimate a slope, intercept or both. It is reasonable to assume that the mutation burden is zero at age zero. Here, we evaluated this assumption by comparing three ways of modeling the relationship between age and SBS5 mutation counts: 1) including patient-specific and population intercept; 2) including patient specific intercept only and 3) constraining the intercept to 0.

Estimating a patient-specific slope and intercept requires more than one datapoint for each patient. Therefore we include here only the 15 patients with >1 phylogenetic branch.

```
multibranch.df <- branch.df3

### free intercepts
muts.per.year.lmer.with.pt.intercepts <- lmer(Num.mutations ~ Age + (1 + Age | Sample), data=multibranch.df, REML=FALSE)
```

```
## Warning in checkConv(attr(opt, "derivs"), opt$par, ctrl = control$checkConv, :
## Model failed to converge with max|grad| = 0.168035 (tol = 0.002, component 1)
```

```
## Warning in checkConv(attr(opt, "derivs"), opt$par, ctrl = control$checkConv, : Model is nearly unidentifiable: large eigenvalue ratio
##  - Rescale variables?
```

```
### patient-specific intercept constrained to 0 
muts.per.year.lmer.with.pop.intercept <- lmer(Num.mutations ~ Age + (0 + Age | Sample), data=multibranch.df, REML=FALSE)
### population and patient-specific intercepts constrained to 0
muts.per.year.lmer <- lmer(Num.mutations ~ 0 + Age + (0 + Age | Sample ), data=multibranch.df, REML=FALSE)

# ANOVA for model differences
pandoc.table(anova(muts.per.year.lmer, 
             muts.per.year.lmer.with.pop.intercept, 
             muts.per.year.lmer.with.pt.intercepts), 
             split.tables = "Inf")
```

```
## 
## -------------------------------------------------------------------------------------------------------------------
##                   &nbsp;                     Df    AIC     BIC    logLik   deviance   Chisq    Chi Df   Pr(>Chisq) 
## ------------------------------------------- ---- ------- ------- -------- ---------- -------- -------- ------------
##           **muts.per.year.lmer**             3    659.2   664.4   -326.6    653.2       NA       NA         NA     
## 
##  **muts.per.year.lmer.with.pop.intercept**   4    661.2   668.1   -326.6    653.2     0.0572     1        0.811    
## 
##  **muts.per.year.lmer.with.pt.intercepts**   6    665.2   675.6   -326.6    653.2       0        2          1      
## -------------------------------------------------------------------------------------------------------------------
```

As shown by the ANOVA results, there was no evidence of improved model fit from including intercepts. Akaike information criterion (AIC) and log likelihood were essencially identical for all models (above). We therefore moved forward with the most parsimonious model: constraining the intercept to 0.

We went on to apply the same LME model in the full dataset. Below we show patient-specific estimates of SBS5 mutation rate (colored slopes) as well as the population average (black line). Points represent observed SBS5 mutational burden in phylogenetic branches and the age at sample acquisition, colored by patient.

```
allsamples.df <- branch.df2

muts.per.year.lmer <- lmer(Num.mutations ~ 0 + Age + (0 + Age | Sample ), data=allsamples.df, REML=FALSE)


allsamples.df$Pt.fitted.num.mutrate <- (fixef(muts.per.year.lmer)["Age"] + 
                                                  ranef(muts.per.year.lmer)$Sample[allsamples.df$Sample,"Age"]) * allsamples.df$Age

# Plotting
ggplot(data = allsamples.df, 
       aes(x=Age,
           y=Num.mutations,
           color=LRISample)) + 
    geom_segment(aes(x=0, 
                     y = 0, 
                     xend=Age, 
                     yend=Pt.fitted.num.mutrate, 
                     colour=LRISample)) +
    geom_abline(intercept=0, 
                slope = fixef(muts.per.year.lmer), size=3) +
    geom_point(shape=16, cex=3) +
    xlim(c(0,80)) + 
    labs(x = "Age (years)",
         y = "Mutations (n)",
         col = "Patient") +
  theme(text = element_text(size=15),
        legend.text=element_text(size=8)) +
    labs(x = 'Age (years)',
         y = 'SBS5 mutations in phylogenetic branch')
```

Below we show the observed mutation and age data for each patient, along with patient-specific slopes (colored lines) and the average slope across the cohort (dashed black line).

```
ggplot(data = allsamples.df, aes(x = Age, y = Num.mutations, color=LRISample)) +
    geom_point(size = 2) +
    geom_segment(aes(x = 0, 
                     y = 0, 
                     xend = Age, 
                     yend = Pt.fitted.num.mutrate)) +
    geom_abline(intercept = 0,
                slope = fixef(muts.per.year.lmer),
                lty = 2) +
    guides(col = F)+
    labs(x = "Age (years)",
         y = "Mutations (n)") +
    facet_wrap(~ LRISample)
```

### Model selection: Model adjustment for potential confounders

Several confounding factors may affect the power of WGS to identify mutations in a tumor sample. Three important factors are sequencing coverage, tumor purity and ploidy (determining the effective sequencing coverage of tumor cells, i.e. allele specific coverage). If these factors introduce bias in the estimation of SBS5 mutation burden, adjusting for them in an LME model may provide a more accurate molecular clock. Furthermore, it is possible that the SBS5 mutation rate accelerates during the late phases of tumor evolution and subclonal diversification. Applying the approach published by Mitchel et al (Cell 2019), we tested the effects of including a quadratic term for age in our LME model, effectively allowing for the mutation rate to parabolically increase with age.

```
# Simple model of SBS5 ~ age
rate_simpl <- lmer(Num.mutations ~  0 + Age + (0 + Age | Sample ), data=allsamples.df, REML=FALSE)

# Model adjusted for the effective tumor coverage (purity * coverage)
rate_adj <- lmer(Num.mutations ~  0 + Age + I((CCF*Coverage)/Ploidy) + (0 + Age | Sample ), data=allsamples.df, REML=FALSE)

# Model including a quadratic age term, allowing for the mutation rate to increase parabolically with age.
rate_adj_square <- lmer(Num.mutations ~  0 + Age + I((CCF*Coverage)/Ploidy) +  I(Age^2) + (0 + Age | Sample ), data=allsamples.df, REML=FALSE)

# Estimate mutation rate per year based on the model adjusted for all covariates
allsamples.df$Pt.adj.fitted.num.mutrate <- (fixef(rate_adj_square)["Age"] + 
                                              ranef(rate_adj_square)$Sample[allsamples.df$Sample,"Age"]) * allsamples.df$Age

rate_models <- list(rate_simpl, rate_adj, rate_adj_square)

pandoc.table(anova(rate_simpl, rate_adj, rate_adj_square), split.tables = "Inf")
```

```
## 
## --------------------------------------------------------------------------------------------
##        &nbsp;          Df   AIC    BIC    logLik   deviance    Chisq    Chi Df   Pr(>Chisq) 
## --------------------- ---- ------ ------ -------- ---------- --------- -------- ------------
##    **rate_simpl**      3    1213   1220   -603.6     1207       NA        NA         NA     
## 
##     **rate_adj**       4    1215   1225   -603.6     1207     0.02621     1        0.8714   
## 
##  **rate_adj_square**   5    1217   1229   -603.5     1207     0.2107      1        0.6462   
## --------------------------------------------------------------------------------------------
```

Compared with the simplest model, where age was the only predictor for SBS5 mutation burden, more complex adjusted models did not result in improved model fit (ANOVA). Indeed, model evaluation parameters such as AIC and log likelihood were almost identical.

Diagnostic plots revealed no clear trends between covariates and residuals (i.e. difference between predicted and observed mutation counts) across models (below).

```
rate_resid_simple <- ggplot(data.frame(age = allsamples.df$Age, residuals = residuals(rate_simpl, type="pearson")), 
                            aes(age, residuals))+
    geom_point()+
    labs(title = "Simple model",
         x = 'Age',
         y = 'residuals')

rate_resid_adj_cov <- ggplot(data.frame(cov = I((allsamples.df$CCF*allsamples.df$Coverage)/allsamples.df$Ploidy), residuals = residuals(rate_adj, type="pearson")), 
                             aes(cov, residuals))+
    geom_point()+
    labs(title = "Adjusted model",
         x = 'Allele-specific coverage',
         y = 'residuals')

rate_resid_squared <- ggplot(data.frame(age = allsamples.df$Age, residuals = residuals(rate_adj_square, type="pearson")), 
                             aes(age^2, residuals))+
    geom_point()+
    labs(title = "Quadratic age model",
         x = 'Age squared',
         y = 'residuals')

ggarrange(rate_resid_simple, 
          rate_resid_adj_cov,
          rate_resid_squared,
          ncol = 3)
```

Shown below are the estimates for median SBS5 mutation rate per year according to the three different models.

```
pandoc.table(data.frame(`Mutation rate per year` = sapply(rate_models, function(x) fixef(x)["Age"]),
                 Model = c("Simple", "Adjusted for allele-specific coverage", "Adjusted for allele-specific coverage and quadratic age term")),
             split.tables   = "Inf")
```

```
## 
## -------------------------------------------------------
##  Mutation.rate.per.year              Model             
## ------------------------ ------------------------------
##          38.76                       Simple            
## 
##          38.14            Adjusted for allele-specific 
##                                     coverage           
## 
##          43.68            Adjusted for allele-specific 
##                            coverage and quadratic age  
##                                       term             
## -------------------------------------------------------
```

For each phylogenetic branch, we went on to compare the predicted mutation counts from the simple and adjusted model, and both models against the observed mutational burden. Black lines are drawn along the diagonals, i.e. where points are expected to cluster if the x and y variables are identical.

```
lme_compare <- ggplot(allsamples.df, aes(x = Pt.fitted.num.mutrate, y = Pt.adj.fitted.num.mutrate, col = LRISample))+
    geom_segment(x = 0, y = 0, xend = 4000, yend = 4000, size = 1, col = "black")+
    scale_x_continuous(limits = c(0,5000))+
    scale_y_continuous(limits = c(0,5000))+
    geom_point(size = 2)+
    guides(col = F)+
    labs(title = "Adjusted vs. simple model",
         x = "Num.mutations ~ 0 + Age +\n(0 + Age | Patient)",
         y = "Num.mutations ~ 0 + Age +\nI((purity*Coverage)/Ploidy) + I(Age^2)\n+ (0 + Age | Patient )",
         col = "Patient")+
    theme(text = element_text(size=12),
        legend.text=element_text(size=8)) 
lme_simple_obs <- ggplot(allsamples.df, aes(x = Pt.fitted.num.mutrate, y = Num.mutations, col = LRISample))+
    geom_segment(x = 0, y = 0, xend = 4000, yend = 4000, size = 1, col = "black")+
    scale_x_continuous(limits = c(0,5000))+
    scale_y_continuous(limits = c(0,5000))+
    geom_point(size = 2)+
    guides(col = F)+
    labs(title = "Observed vs. simple",
         x = "Num.mutations ~ 0 + Age\n+ (0 + Age | Patient)",
         y = "Observed Num.mutations",
         col = "Patient")+
    theme(text = element_text(size=12),
        legend.text=element_text(size=8)) 

lme_adj_obs <- ggplot(allsamples.df, aes(x = Pt.adj.fitted.num.mutrate, y = Num.mutations, col = LRISample))+
    geom_segment(x = 0, y = 0, xend = 4000, yend = 4000, size = 1, col = "black")+
    scale_x_continuous(limits = c(0,5000))+
    scale_y_continuous(limits = c(0,5000))+
    geom_point(size = 2)+
    guides(col = guide_legend(ncol=8))+
    labs(title = "Observed vs. adjusted",
         x = "Num.mutations ~ 0 + Age +\nI((purity*Coverage)/Ploidy)+  I(Age^2)\n+ (0 + Age | Patient )",
         y = "Observed Num.mutations",
         col = "Patient")+
    theme(text = element_text(size=12),
         legend.text=element_text(size=10)) 

ggarrange(lme_compare, lme_simple_obs, lme_adj_obs, common.legend = T, legend = "bottom", nrow = 1)
```

Below, we show the estimated SBS5 mutation rate with standard deviation for each patient, as determined by the different LME models. The average SBS5 mutation rate is shown as a horizontal black line. Dots with error-bars were colored by the disease stage: red for patients who entered the study as smoldering multiple myeloma and then progressed; blue for patients who only had one sample with newly diagnosed myeloma; and green for patients who relapsed.

```
# Variable setup

mutrate_patients_list <- allsamples.df %>%
  group_by(Sample) %>%
  mutate(stage = factor(ifelse(any("Smoldering MM" %in% Stage), "Smoldering MM", 
                             ifelse(any("Relapsed MM" %in% Stage), "Relapsed MM", "Newly diagnosed MM")),
                        levels = c("Smoldering MM", "Newly diagnosed MM", "Relapsed MM"))) %>%
  as.data.frame() %>%
  distinct(Sample, stage)

# SIMPLE MODEL

# obtaining the population estimate for mutation rate
mutrate_patients_simple <- mutrate_patients_list
mutrate_patients_simple$fixed_effect <- fixef(rate_simpl)

# simulating the uncertainty in patient-specific adjustment factor for mutation rate (random effect)
random_effect_sim <- REsim(rate_simpl, n.sims = 1000)
random_effect_sim <- random_effect_sim[c(2,4,6)]
names(random_effect_sim) <- c("Sample", "ranef_mean", "ranef_sd")

mutrate_patients_simple <- mutrate_patients_simple %>%
    left_join(random_effect_sim, by = "Sample") %>%
    mutate(sd_upper = fixed_effect + ranef_mean + ranef_sd,
           pt_est = fixed_effect + ranef_mean,
           sd_lower = fixed_effect + ranef_mean - ranef_sd) %>%
    arrange(pt_est)

# ADJUSTED MODEL

# obtaining the population estimate for mutation rate
mutrate_patients_adj <- mutrate_patients_list
mutrate_patients_adj$fixed_effect <- fixef(rate_adj)["Age"]

# simulating the uncertainty in patient-specific adjustment factor for mutation rate (random effect)
random_effect_sim <- REsim(rate_adj, n.sims = 1000)
random_effect_sim <- random_effect_sim[c(2,4,6)]
names(random_effect_sim) <- c("Sample", "ranef_mean", "ranef_sd")

mutrate_patients_adj <- mutrate_patients_adj %>%
    left_join(random_effect_sim, by = "Sample") %>%
    mutate(sd_upper = fixed_effect + ranef_mean + ranef_sd,
           pt_est = fixed_effect + ranef_mean,
           sd_lower = fixed_effect + ranef_mean - ranef_sd) %>%
    arrange(pt_est)

# QUADRATIC MODEL

# obtaining the population estimate for mutation rate
mutrate_patients_adj_square <- mutrate_patients_list
mutrate_patients_adj_square$fixed_effect <- fixef(rate_adj_square)["Age"]

# simulating the uncertainty in patient-specific adjustment factor for mutation rate (random effect)
random_effect_sim <- REsim(rate_adj_square, n.sims = 1000)
random_effect_sim <- random_effect_sim[c(2,4,6)]
names(random_effect_sim) <- c("Sample", "ranef_mean", "ranef_sd")

mutrate_patients_adj_square <- mutrate_patients_adj_square %>%
    left_join(random_effect_sim, by = "Sample") %>%
    mutate(sd_upper = fixed_effect + ranef_mean + ranef_sd,
           pt_est = fixed_effect + ranef_mean,
           sd_lower = fixed_effect + ranef_mean - ranef_sd) %>%
    arrange(pt_est)
```

```
# PLOTTING

mutrate_simple_plot <- ggplot(data = mutrate_patients_simple, aes(col = stage))+
    geom_point(aes(reorder(Sample, pt_est), pt_est), size = 3)+
    geom_errorbar(aes(x = reorder(Sample, pt_est), ymin = sd_lower, ymax = sd_upper))+
    geom_hline(yintercept = fixef(rate_simpl), size = 1, col = "black")+
    scale_color_brewer(palette = "Set1")+
    scale_y_continuous(limits = c(0,70))+
    labs(title = "Simple model",
         y = "SBS5 mutation rate",
         col = "Disease stage")+
    theme(axis.title.x = element_blank(),
          axis.text.x = rotatedAxisElementText(90, "top"),
          axis.text = element_text(size = 12))

mutrate_adj_plot <- ggplot(data = mutrate_patients_adj, aes(col = stage))+
    geom_point(aes(reorder(Sample, pt_est), pt_est), size = 3)+
    geom_errorbar(aes(x = reorder(Sample, pt_est), ymin = sd_lower, ymax = sd_upper))+
    geom_hline(yintercept = fixef(rate_adj)["Age"], size = 1, col = "black")+
    scale_color_brewer(palette = "Set1")+
    scale_y_continuous(limits = c(0,70))+
    labs(title = "Adjusted model",
         y = "SBS5 mutation rate",
         col = "Disease stage")+
    theme(axis.title.x = element_blank(),
          axis.text.x = rotatedAxisElementText(90, "top"),
          axis.text = element_text(size = 12))

mutrate_adj_square_plot <- ggplot(data = mutrate_patients_adj_square, aes(col = stage))+
    geom_point(aes(reorder(Sample, pt_est), pt_est), size = 3)+
    geom_errorbar(aes(x = reorder(Sample, pt_est), ymin = sd_lower, ymax = sd_upper))+
    geom_hline(yintercept = fixef(rate_adj_square)["Age"], size = 1, col = "black")+
    scale_color_brewer(palette = "Set1")+
    scale_y_continuous(limits = c(0,70))+
    labs(title = "Quadratic age model",
         y = "SBS5 mutation rate",
         col = "Disease stage")+
    theme(axis.title.x = element_blank(),
          axis.text.x = rotatedAxisElementText(90, "top"),
          axis.text = element_text(size = 12))

ggarrange(mutrate_simple_plot, mutrate_adj_plot, mutrate_adj_square_plot, common.legend = T, legend = "bottom", nrow = 3)
```

Patients at different stages of disease did not differ with respect to the estimated SBS5 mutation rate, as confirmed below using a pairwise wilcoxon test.

```
pairwise.wilcox.test(mutrate_patients_simple$pt_est, mutrate_patients_simple$stage)
```

```
## 
##  Pairwise comparisons using Wilcoxon rank sum test 
## 
## data:  mutrate_patients_simple$pt_est and mutrate_patients_simple$stage 
## 
##                    Smoldering MM Newly diagnosed MM
## Newly diagnosed MM 1             -                 
## Relapsed MM        1             1                 
## 
## P value adjustment method: holm
```

```
#pairwise.wilcox.test(mutrate_patients_adj$pt_est, mutrate_patients_adj$stage)
#pairwise.wilcox.test(mutrate_patients_adj_square$pt_est, mutrate_patients_adj_square$stage)
```

Based on the above analysis, we found no benefit from including adjustment variables in the LME model for SBS5 mutation rate. Moreover, there was no evidence that disease stage at the time of sampling affected the mutation rate estimates. We therefore went on to use the most parsimonious (i.e. unadjusted) models in the main analysis.

Below, we show the average SBS5 mutation rate with between-patient variation estimated from the simple LME model.

```
# cohort average rate
fixef_est <- as.numeric(fixef(rate_simpl))

std_err_fixef <- summary(rate_simpl)$coefficients[1,2]

# 95 % CI for the cohort average
upperCI <-  fixef_est + 1.96*std_err_fixef
lowerCI <-  fixef_est  - 1.96*std_err_fixef

# standard deviation of between-patient differences
between_pts_sd <- sd(mutrate_patients_simple$pt_est)

# Between-patient standard deviation as percent of the average SBS5 mutation rate
between_pts_percent <- between_pts_sd/fixef_est*100

paste0("Average SBS5 mutation rate: ", round(fixef_est,2), " (", round(lowerCI, 2), "-", round(upperCI, 2), ")")
```

```
## [1] "Average SBS5 mutation rate: 38.76 (35.74-41.78)"
```

```
paste0("Between-patient standard deviation: ", round(between_pts_sd, 2), " (", round(between_pts_percent, 2), " %)")
```

```
## [1] "Between-patient standard deviation: 7.09 (18.3 %)"
```

## Absolute timing of landmark events

### Timing the MRCA emergence

MRCA estimation is vulnerable to incorrect reconstruction of the phylogenetic tree, particularly when branches are incorrectly assigned as part of the trunk. Therefore we restricted the analysis of MRCA to patients with multiple samples, where phylogenetic tree reconstruction is more accurate. We followed the same procedure for MRCA estimation as previously reported by our group (Mitchell et al, Cell, 2018), predicting age at MRCA emergence from SBS5 mutational burden in the trunk, and applying a bootstrapping approach to estimate 95 % confidence intervals.

```
### Helper functions for MRCA analysis

# Function to estimate the time of MRCA
estimateMRCA <- function(input.df, lmer.model){
    
    # Bootstrapping function for MRCA
    mySumm <- function(.) {
        predict(., newdata=mrca.df, re.form=NULL)
    }
    
    # Collapse bootstrap into median, 95% prediction interval (PI)
    sumBoot <- function(merBoot) {
        return(
            data.frame(fit = apply(merBoot$t, 2, function(x) as.numeric(quantile(x, probs=.5, na.rm=TRUE))),
                       lwr = apply(merBoot$t, 2, function(x) as.numeric(quantile(x, probs=.025, na.rm=TRUE))),
                       upr = apply(merBoot$t, 2, function(x) as.numeric(quantile(x, probs=.975, na.rm=TRUE)))
            )
        )
    }
    
    # setup variable
    mrca.df <- input.df
    mrca.df <- mrca.df[!duplicated(mrca.df$Sample),]
    
    # predict the age of MCRA
    mrca.df$MRCA.pred.age <- predict(lmer.model, newdata = mrca.df) 
    
    # Now generate 95% CIs on the predictions for MRCA timing
    # Note that CIs for lme models are challenging - bootstrapping appears to be most robust
    ## bootMer = Perform model-based (Semi-)parametric bootstrap for mixed models.
    boot1 <- bootMer(lmer.model, mySumm, nsim=1000, use.u=FALSE, type="parametric")
    
    PI.boot1 <- sumBoot(boot1)
    PI.boot1 <- PI.boot1[complete.cases(PI.boot1),]
    mrca.df2 <- mrca.df[complete.cases(mrca.df$MRCA.pred.age),]
    PI.boot1$Age <- mrca.df2$Age
    PI.boot1$pred.fit <- mrca.df2$MRCA.pred.age
    PI.boot1$Time.lag <- PI.boot1$Age - PI.boot1$fit
    PI.boot1$Time.lag[PI.boot1$Time.lag < 0] <- 0 # Time lag < 0 are set to 0
    PI.boot1$lag.lwr <- PI.boot1$Age - PI.boot1$upr
    PI.boot1$lag.lwr[PI.boot1$lag.lwr < 0] <- 0
    PI.boot1$lag.upr <- PI.boot1$Age - PI.boot1$lwr
    PI.boot1$lag.upr[PI.boot1$lag.upr < 0] <- 0
    PI.boot1$ID <- mrca.df2$Sample
    PI.boot1$LRIID <- mrca.df2$LRISample
    PI.boot1 <- PI.boot1[order(PI.boot1$Age),]
    PI.boot1$LRIID<- as.character( PI.boot1$LRIID)
    # order by time lag
    PI.boot1 <- PI.boot1[order(PI.boot1$Time.lag),]
    
    return(PI.boot1)
}

# plotting function for MRCA
plotMRCA <- function(input.df){
    
    PI.boot1 <- input.df
    
    par(mfrow = c(1, 1),
        xpd = T,
        mar = c(7, 5, 5, 5))
    
    plot(
        PI.boot1$Time.lag,
        1:nrow(PI.boot1),
        pch = 16,
        xlim = c(-10, max(PI.boot1$lag.upr)),
        axes = FALSE,
        xlab = "Estimated time between MRCA and first sample collection (years)",
        ylab = "",
        col = "dodgerblue3",
        cex = 1,
        cex.lab = 1
    )
    
    segments(
        x0 = PI.boot1$lag.lwr,
        x1 = PI.boot1$lag.upr,
        y0 = 1:nrow(PI.boot1),
        col = "dodgerblue2"
    )
    
    par(new = T)
    plot(
        PI.boot1$Time.lag,
        1:nrow(PI.boot1),
        pch = 16,
        xlim = c(-10, max(PI.boot1$lag.upr)),
        axes = FALSE,
        xlab = "",
        ylab = "",
        col = "dodgerblue3",
        cex = 1.7
    )
    
    
    axis(
        side = 1,
        at = (0:5) * 10,
        labels = (0:5) * 10,
        cex.axis = 2
    )
    # top axis
    axis(
        side = 3,
        at = (0:5) * 10,
        labels = (0:5) * 10,
        cex.axis = 2
    )
    
    grid(ny = (0:5) * 10, nx = NULL)
    
    
    text(
        x = PI.boot1$lag.lwr - 1,
        y = 1:nrow(PI.boot1),
        labels = PI.boot1$LRIID,
        adj = 1,
        cex = 1.6
    )
}
```

```
# Train LME model to predict age from mutational burden

allsamples.df$Scaled.num.muts <- scale(allsamples.df$Num.mutations, center=FALSE)  # divide by root mean square
scale.factor.muts <- attr(allsamples.df$Scaled.num.muts, which = "scaled:scale") # root mean square value

age_lmer <- lmer(Age ~ 0 + Scaled.num.muts + (0 + Scaled.num.muts | Sample), data=allsamples.df)

# Dataset for MRCA predicion where the Scaled.num.muts variable is set to the scaled number of SBS5 mutations in the trunk
to.mrca.all <- with(allsamples.df, 
                         data.frame(Sample=Sample, 
                              LRISample=LRISample,
                              Scaled.num.muts=Num.clonal / scale.factor.muts, #### scaled number of mutation in the trunk
                              Age=Age))

# Run pre-defined function to estimate MRCA
allsamples.mrca <- estimateMRCA(to.mrca.all, age_lmer)

multisample <- c("PD26423", "PD26403", "PD26419", "PD26435", "PD26418", "PD26401",
                 "PD26408", "PD26416", "PD26424", "PD26402", "PD26422",
                 "PD26411", "PD26409", "PD26432", "PD26400", "PD26420", "PD26425", 
                 "PD26407", "PD26405", "PD26404", "PD26427", "PD26412", "PD26406", 
                 "PD26415", "PD26428")

allsamples.mrca.sub <- allsamples.mrca[allsamples.mrca$LRIID %in% multisample,]
```

```
# MRCA timing -- plotting only patients with multiple samples
plotMRCA(allsamples.mrca.sub)
```

### Timing of multi-gain events

Setting up the data for absolute timing analysis of large gains occurring within distinct time-windows: 1) Define the somatic mutations involved in each time-window; 2) determine the mutational signatures active in each tine-window; 3) estimate mutational signature contributions in duplicated and non-duplicated mutations for each time-window using mmsig (https://github.com/evenrus/mmsig); 4) estimate the SBS5-based molecular timing of each multi-gain event (separately for the point estimate, 2.5th and 97.5th percentiles of SBS5 mutation burden); 5) generate final summary of each time window with molecular time, SBS5 mutational burden and total gain size.

```
### Helper function for timing of gains

# Estimate absolute timing of gains
estimateGains <- function(clin, time_windows_mol_time, allsamples.mrca, muts.per.year.lmer){
    
    # Estimating patient-specific mutation rates (mut/year)
    abs.time <- time_windows_mol_time %>%
        left_join(clin %>%
                      dplyr::select(sample_ID, Age)%>%
                      dplyr::rename(sample = "sample_ID") %>%
                      mutate(sample = as.character(sample)),
                  by = 'sample') %>%
        dplyr::select(patient, time_window, size, variable, Age, SBS5_CN2_count, mol_time_est, mol_time_2.5, mol_time_97.5) %>%
        dplyr::rename(Sample = 'patient',
                      `2.5%` = mol_time_2.5,
                      `97.5%` = mol_time_97.5,
                      mol_time = mol_time_est) %>%
        mutate(size = size/1000000000) # scale to gigabases
    
    abs.time$Est.mut.rate.per.Gb.per.year <- (fixef(muts.per.year.lmer)["Age"] + 
                                                  ranef(muts.per.year.lmer)$Sample[abs.time$Sample,"Age"]) / 6
    
    abs.time <- abs.time[!is.na(abs.time$Est.mut.rate.per.Gb.per.year),]
    
    abs.time$age_gain<- abs.time$SBS5_CN2_count/(abs.time$size* abs.time$Est.mut.rate.per.Gb.per.year)
    
    if(is.null(allsamples.mrca)){
        # If no MRCA data
        abs.time <- abs.time %>%
            mutate(MRCA.est = NA)
    } else {
        mrca.mod <- allsamples.mrca %>%
            rowwise() %>%
            mutate(Sample = as.character(LRIID), 
                   # Predicted MRCA earlier than first sample are adjusted to the time of first sample
                   # An offset of -0.5 is added for plotting purposes.
                   MRCA.est = ifelse(Age - fit > 0, fit, Age-0.5)) %>% 
            ungroup() %>%
            dplyr::select(Sample, MRCA.est)
        
        abs.time <- abs.time %>%
            left_join(mrca.mod, by = 'Sample')
    }
    abs.time <- abs.time[!duplicated(abs.time),]
    return(abs.time)
}

# absolute time plots
plotGains <- function(abs.time){
    par(mar=c(10,10,5,17), xpd=T)
    sample_list <- unique(abs.time$Sample)
    max_age <- max(abs.time$Age)+5
    
    for(i in (1:length(sample_list))){
        s <- sample_list[i]
        sub <- abs.time[abs.time$Sample == s,]
        
        if(nrow(sub)==1 & sub$time_window[1] == "first"){
            plot(sub$age_gain,i, pch=20, xlim=c(-10,(max_age)), ylim=c(0,length(sample_list)), col="forestgreen",
                 axes=FALSE, xlab="Estimated time Chrom Gain (years)", ylab="", cex=2)
            
            segments(x0 = sub$age_gain*(sub$`2.5%`/sub$mol_time), 
                     x1 = sub$age_gain*(sub$`97.5%`/sub$mol_time),
                     y0 = i, col="forestgreen")
            
            par(new=T)
            plot(sub$Age,i, pch=16, col="brown3",  xlab="", ylab="",ylim=c(0,length(sample_list)),
                 axes=FALSE,  , xlim=c(-10,(max_age)), cex=2)
            
            par(new=T)
            plot(sub$MRCA.est, i, pch=16, col="blue",  xlab="", ylab="",ylim=c(0,length(sample_list)),
                 axes=FALSE,  , xlim=c(-10,(max_age)), cex=2)
            
        } else {
            if(nrow(sub)==1 & sub$time_window[1] == "second"){
                
                plot(sub$age_gain,i, pch=20, xlim=c(-10,(max_age)), ylim=c(0,length(sample_list)), col="darkolivegreen3",
                     axes=FALSE, xlab="Estimated time Chrom Gain (years)", ylab="", cex=2)
                
                segments(x0 = sub$age_gain*(sub$`2.5%`/sub$mol_time), 
                         x1 = sub$age_gain*(sub$`97.5%`/sub$mol_time),
                         y0 = i, col="darkolivegreen3")
                
                par(new=T)
                plot(sub$Age,i, pch=16, col="brown3",  xlab="", ylab="",ylim=c(0,length(sample_list)),
                     axes=FALSE,  , xlim=c(-10,(max_age)), cex=2)
                
                par(new=T)
                plot(sub$MRCA.est, i, pch=16, col="blue",  xlab="", ylab="",ylim=c(0,length(sample_list)),
                     axes=FALSE,  , xlim=c(-10,(max_age)), cex=2)
            } else {
                sub<- sub[order(sub$age_gain),] 
                plot(sub$age_gain,rep(i, nrow(sub)), pch=20, xlim=c(-10,(max_age)), ylim=c(0,length(sample_list)), 
                     axes=FALSE, xlab="Estimated time Chrom Gain (years)", ylab="", cex=2, col=c("forestgreen","darkolivegreen3"))
                
                segments(x0 = sub$age_gain[1]*(sub$`2.5%`[1]/sub$mol_time[1]), 
                         x1 = sub$age_gain[1]*(sub$`97.5%`[1]/sub$mol_time[1]),
                         y0 = i, col="forestgreen")
                
                segments(x0 = sub$age_gain[2]*(sub$`2.5%`[2]/sub$mol_time[2]), 
                         x1 = sub$age_gain[2]*(sub$`97.5%`[2]/sub$mol_time[2]),
                         y0 = i, col="darkolivegreen4")
                
                par(new=T)
                plot(unique(sub$Age),i, pch=16, col="brown3",  xlab="", ylab="",ylim=c(0,length(sample_list)),
                     axes=FALSE,  xlim=c(-10,(max_age)), cex=2)
                
                par(new=T)
                plot(unique(sub$MRCA.est), i, pch=16, col="blue",  xlab="", ylab="",ylim=c(0,length(sample_list)),
                     axes=FALSE,  xlim=c(-10,(max_age)), cex=2)
            }
        }
        par(new=T)
    }
    axis(side=1, at=seq(0,80, by=10), labels=seq(0,80, by=10), cex.axis=2)
    text(x = rep(0, length(sample_list)),
         y = 1:length(sample_list), labels = sample_list, cex=1.5,offset =F, adj = c(1,0) )
    
    legend("topright",legend=c("Sampling","MRCA","1st Time Window","2nd Time Window"),bty="n", pch=16, 
           col=c("brown3","blue","forestgreen","darkolivegreen4"),
           cex=1.8, pt.cex=1.8, inset=c(-0.45,0),x.intersp = 1, y.intersp = 1)
    
}
```

```
### 1) Define the somatic mutations involved in each time-window

# define mutational profiles of each time-window
muts_order <- mut_cnv_order[,c("sample","chr","pos","ref","alt")]

mut_catalogue <- mut.to.sigs.input(mut.ref = muts_order,
                                  sample.id = "sample",
                                  chr = "chr",
                                  pos = "pos",
                                  ref = "ref",
                                  alt = "alt",
                                  bsg = BSgenome.Hsapiens.UCSC.hg19)
```

```
## Warning in mut.to.sigs.input(mut.ref = muts_order, sample.id = "sample", : Some samples have fewer than 50 mutations:
##   V0D583_first_2, V0D56W_second_1, V0D56W_second_2, V0D57M_second_1, V0D57M_second_2, PD26435c_first_2, V0D58T_second_1, V0D58T_second_2, V0D583_second_2
```

```
mut_catalogue <- data.frame(t(mut_catalogue))

### 2) assign the mutational signatures active in each tine-window
sample.sigt.profs.final <- list()
for(i in 1:ncol(mut_catalogue)){
    s <- names(mut_catalogue)[i]
    s_split <- unlist(stri_split_fixed(s, "_", n = 2))[1]
    s_sigt.profs <- sample.sigt.profs[[s_split]]
    sample.sigt.profs.final[[s]] <- s_sigt.profs
}

### 3) estimate mutational signature contributions in duplicated and non-duplicated mutations for each time-window

# If data is not available in working directory, runs mutational signature fitting. Otherwise loads prepared data.
if(!file.exists("data/bootstrap1000_SBS_prepost.csv")){
    mutSigsSummary <- mm_fit_signatures(muts.input = mut_catalogue, 
                                        sig.input = sig_ref,
                                        input.format = "classes",
                                        sample.sigt.profs = sample.sigt.profs.final, 
                                        bootstrap = TRUE,
                                        iterations = 1000)$bootstrap
    
    write.csv(mutSigsSummary, "data/bootstrap1000_SBS_prepost.csv", row.names = F)
} else {
    mutSigsSummary <- read.csv("data/bootstrap1000_SBS_prepost.csv", stringsAsFactors = F)
}

### 4) estimate the SBS5-based molecular timing of each multi-gain event (separately for the point estimate, 2.5th and 97.5th percentiles of SBS5 mutation burden).

SBS5_mol_time <- mutSigsSummary %>%
    filter(signature == "SBS5") %>%
    left_join(data.frame(total = colSums(mut_catalogue)) %>% # Adding total mutation counts per sample
                  rownames_to_column("sample"), 
              by = 'sample') %>%
    rowwise() %>%
    mutate(CI025 = as.integer(CI025 * total),
           CI975 = as.integer(CI975 * total),
           estimate = as.integer(estimate * total)) %>%
    as.data.frame() %>%
    dplyr::select(-signature, -total, -mean) %>%
    separate(col = sample, into = c("sample", "time_window", "pre_post"), sep = "_") %>%
    melt(id.vars = c("sample", "time_window", "pre_post")) %>%
    group_by(sample, time_window, variable) %>%
    summarise(SBS5_CN1_count = value[which(pre_post == 1)],
              SBS5_CN2_count = value[which(pre_post == 2)],
              mol_time = paste(triploid.2.1.est(SBS5_CN1_count+1, SBS5_CN2_count+1, iter=iter)$pest, collapse = "_")) %>% # Run molecular time
    separate(col = mol_time, into = c("mol_time_est", "mol_time_2.5", "mol_time_97.5"), sep = "_", convert = TRUE) %>%
    as.data.frame()

### 5) generate final summary file of each time window with molecular time, SBS5 mutational burden and total gain size.

time_windows_mol_time <- mut_cnv_order %>%
    mutate(sample = gsub("_.*", "", sample)) %>%
    dplyr::rename(time_window = 'order') %>%
    distinct(sample, time_window, segment_size) %>%
    group_by(sample, time_window) %>%
    summarise(size = sum(segment_size)) %>%
    as.data.frame() %>%
    right_join(SBS5_mol_time, by = c("sample", "time_window")) %>%
    left_join(tree %>%
                  dplyr::select(sample_ID, patient) %>%
                  dplyr::rename(sample = 'sample_ID'), by = 'sample')

# Exclude time windows with < 100 mutations pre/post gain (due to uncertainty in timing estimates)
exclude_low_mut<- time_windows_mol_time[(time_windows_mol_time$SBS5_CN1_count + time_windows_mol_time$SBS5_CN2_count) <100 & time_windows_mol_time$variable=="estimate",]

time_windows_mol_time <- time_windows_mol_time[! paste(time_windows_mol_time$sample, time_windows_mol_time$time_window) %in% 
                                                 paste(exclude_low_mut$sample, exclude_low_mut$time_window),]

# Remove duplicates
time_windows_mol_time <- time_windows_mol_time[!duplicated(time_windows_mol_time),]
```

Plotting duplicated and non-duplicated SBS5 mutations and molecular time estimates for the first gain.

```
SBS5_prepost_toplot <- SBS5_mol_time %>%
    filter(time_window == "first") %>%
    dplyr::select(sample, variable, SBS5_CN1_count, SBS5_CN2_count) %>%
    dplyr::rename(est = 'variable') %>%
    melt(id.vars = c('sample', 'est')) %>%
    spread(est, value) %>%
    mutate(variable = ifelse(variable == "SBS5_CN1_count", "Non-duplicated", "Duplicated"))

SBS5_plot <- ggplot()+
    geom_point(data = SBS5_prepost_toplot, 
               aes(sample, estimate, col = variable), size = 2)+
    geom_segment(data = SBS5_prepost_toplot, 
                 aes(x = sample, xend = sample, y = CI025, yend = CI975, col = variable), size = 1)+
    scale_color_brewer(palette = 'Set2')+
    labs(x = 'Sample',
         y = 'SBS5 mutation count')+
    theme(text = element_text(size = 12),
          axis.text.x = element_blank(),
          axis.title.x = element_blank(),
          axis.ticks.x = element_blank(),
          legend.position = 'none',
          plot.margin = unit(c(5.5, 5.5, 5.5, 5.5), "points"))+
    facet_grid(variable~., scale = "free_y")

# Plotting SBS5 pre/post the first gain

moltime_prepost_toplot <- SBS5_mol_time %>%
    filter(time_window == "first") %>%
    dplyr::select(sample, variable, mol_time_est, mol_time_2.5, mol_time_97.5) %>%
    mutate(variable = factor(ifelse(variable == "CI025", "2.5 %", 
                                    ifelse(variable == "CI975", "97.5 %", "estimate")),
                             levels = c("2.5 %", "estimate", "97.5 %")))

moltime_plot <- ggplot()+
    geom_point(data = moltime_prepost_toplot, 
               aes(variable, mol_time_est, col = variable), size = 2)+
    geom_segment(data = moltime_prepost_toplot, 
                 aes(x = variable, xend = variable, y = mol_time_2.5, yend = mol_time_97.5, col = variable), size = 1)+
    scale_color_brewer(palette = 'Set1')+
    labs(x = 'Sample',
         y = 'Molecular time',
         col = "Variability in mutational signature fitting")+
    theme(text = element_text(size = 12),
          axis.text.x = element_blank(),
          strip.text.x = element_text(angle = 90),
          axis.title.x = element_blank(),
          axis.ticks.x = element_blank(),
          strip.background = element_blank(),
          legend.position = 'bottom',
          plot.margin = unit(c(5.5, 25, 5.5, 10), "points"))+
    facet_grid(~sample, switch="both")

ggarrange(SBS5_plot, 
          moltime_plot, 
          heights = c(1, 1),
          nrow = 2)
```

We went on to evaluate whether the size of multi-gain events affected the timing estimates, but this was not the gase, as shown below.

```
# molecular time and gain size

annotate_figure(ggplotRegression(lm(size ~ mol_time_est, data = filter(time_windows_mol_time, time_window == 'first', variable == 'estimate')), 
                                 NULL, "Molecular time"), 
                left = "Total size of first multi-gain event (nt)")
```

Below we show absolute timing of gains in the first (dark green) and second (light green) time-windows based on the point estimate for SBS5 mutation burden. Bootstrapping confidence intervals are shown for the molecular time estimate with that SBS5 mutational burden. MRCA is shown for patients with branching evolution.

```
muts.per.year.lmer <- lmer(Num.mutations ~ 0 + Age + (Age + 0 | Sample), data=allsamples.df, REML=FALSE)

multigains.simple <-  estimateGains(clin = clin, 
                                    time_windows_mol_time = time_windows_mol_time, 
                                    allsamples.mrca = allsamples.mrca.sub, 
                                    muts.per.year.lmer = muts.per.year.lmer)

plotGains(filter(multigains.simple, variable == 'estimate'))
```

## Impact of uncertainty in mutational signature fitting on timing of landmark events

To account for the uncertainty in mutational signature fitting, we show here the timing estimates for landmark events based on the 2.5th and 97.5th percentiles of SBS5 mutational burden.

### Timing the MRCA

```
# Obtaining 95 % CI for mutations in the trunk
trunks <- read.delim("./data/trunk_96classes.txt")

# Set up mutational signatures for the trunks
sig_trunks <- gsub("[a-z]$", "", names(sample.sigt.profs))
sig_trunks_unique <- unique(sig_trunks)
trunk.sigt.profs <- list()
for(i in 1:length(sig_trunks_unique)){
  tr <- sig_trunks_unique[i]
  sigs <- unique(unlist(sample.sigt.profs[which(sig_trunks == tr)]))
  trunk.sigt.profs[[i]] <- sigs
  names(trunk.sigt.profs)[i] <- tr
}

# If data is not available in working directory, runs mutational signature fitting. Otherwise loads prepared data.
if(!file.exists("data/bootstrap1000_SBS_trunks.csv")){
    sigTrunks <- mm_fit_signatures(muts.input = trunks, 
                                        sig.input = sig_ref,
                                        input.format = "classes",
                                        sample.sigt.profs = trunk.sigt.profs, 
                                        bootstrap = TRUE,
                                        iterations = 1000)$bootstrap
    
    write.csv(sigTrunks, "data/bootstrap1000_SBS_trunks.csv", row.names = F)
} else {
    sigTrunks <- read.csv("data/bootstrap1000_SBS_trunks.csv", stringsAsFactors = F)
}

trunkMuts <- data.frame(total = colSums(trunks)) %>%
  rownames_to_column(var = "sample") %>%
  left_join(sigTrunks, by = "sample") %>%
  filter(signature == "SBS5") %>%
  dplyr::select(-mean, -signature) %>%
  melt(id.vars = c("sample", "total"), variable.name = "est", value.name = "Num.clonal") %>%
  mutate(Num.clonal = as.integer(total*Num.clonal)) %>%
  dplyr::rename(Sample = "sample") %>%
  left_join(allsamples.df %>%
              dplyr::select(Sample, Num.mutations, Age, CCF, Coverage),
            by = "Sample") %>%
  filter(!is.na(Num.mutations)) %>%
  mutate(LRISample = Sample,
         Scaled.num.muts = Num.clonal / scale.factor.muts)   # Scaling the trunkal mutation burden

# MRCA estimation
allsamples.mrca.est <- estimateMRCA(filter(trunkMuts, est == "estimate"), age_lmer)
allsamples.mrca.2.5 <- estimateMRCA(filter(trunkMuts, est == "CI025"), age_lmer)
allsamples.mrca.97.5 <- estimateMRCA(filter(trunkMuts, est == "CI975"), age_lmer)

mrca.comb.sub <- rbind(allsamples.mrca.est %>%
                                    mutate(fitting = "estimate"),
                                  allsamples.mrca.2.5 %>%
                                    mutate(fitting = "CI025"),
                                  allsamples.mrca.97.5 %>%
                                    mutate(fitting = "CI975")) %>%
                              filter(LRIID %in% multisample)
```

Below we show how uncertainty in mutational signature fitting influences the lag-time estimates from MRCA to sample acquisition. Point estimates with 95 % CI for SBS5 mutational burden was estimated using mmsig (colors). For each mutational signature estimate, prediction of lag-time from MRCA to sampling was performed as described above and presented as point estimate (points) with 95 % CI (vertical lines). Patient PD26428 was an outlier in this analysis due to a very short trunk of the phylogenetic tree, with predicted MRCA in the first decade of life.

```
# Plotting
ggplot(mrca.comb.sub)+
    geom_point(aes(fitting, Time.lag, col = fitting), size = 2)+
    geom_segment(aes(x = fitting, xend = fitting, y = lag.lwr, yend = lag.upr, col = fitting), size = 1)+
    scale_color_brewer(palette = 'Set1')+ 
    labs(y = 'Time-lag from MRCA to sampling',
         col = "Variability in mutational signature fitting")+
    theme(text = element_text(size = 12),
          axis.text.x = element_blank(),
          axis.ticks.x = element_blank(),
          axis.title.x = element_blank(),
          strip.text.x = rotatedAxisElementText(90, "top"),
          strip.background = element_blank(),
          legend.position = 'bottom')+
    facet_grid(~LRIID, switch = 'x')
```

### Timing the first multi-gain event

```
# Combining the models
multigains.sig <- multigains.simple %>%
    dplyr::rename(fitting = 'variable') %>%
    filter(time_window == 'first') %>%
    rowwise() %>%
    mutate(age_gain_lower = age_gain*(`2.5%`/mol_time),
           age_gain_upper = age_gain*(`97.5%`/mol_time),
           gain_lag_time = Age - age_gain,
           fitting = factor(ifelse(fitting == "CI025", "2.5 %",
                                   ifelse(fitting == "CI975", "97.5 %", "estimate")),
                            levels = c("2.5 %", "estimate", "97.5 %"))) %>%
    as.data.frame() %>%
    dplyr::select(Sample, fitting, age_gain, age_gain_lower, age_gain_upper)
```

Below we show how uncertainty in mutational signature fitting influences timing of the first multi-gain event. Point estimates with 95 % CI for SBS5 mutational burden was estimated using mmsig (colors). For each mutational signature estimate, the age at acquisiton of the first multi-gain event with 95 % CI was estimated as described above.

```
# Plotting
ggplot(multigains.sig)+
    geom_hline(yintercept = 30, linetype = 3, size = 1)+
    geom_point(aes(fitting, age_gain, col = fitting), size = 2)+
    geom_segment(aes(x = fitting, xend = fitting, y = age_gain_lower, yend = age_gain_upper, col = fitting), size = 1)+
    scale_color_brewer(palette = 'Set1')+
    labs(y = 'Age at the first multi-gain event',
         col = "Variability in mutational signature fitting")+
    theme(text = element_text(size = 12),
          axis.text.x = element_blank(),
          axis.ticks.x = element_blank(),
          axis.title.x = element_blank(),
          strip.text.x = rotatedAxisElementText(90, "x"),
          strip.background = element_blank(),
          legend.position = 'bottom')+
    facet_grid(~Sample)
```

## Potential bleeding of mutational signatures between SBS-MM1 and SBS5: Impact on timing of the first multi-gain event

The mutational profiles of SBS5 and SBS-MM1 have some similarities (Figure 1A). Consequently, mutations induced by SBS-MM1 may be erroniously assigned to SBS5, or vice versa. Both of these scenarios may affect the estimated mutation rates in patients with SBS-MM1. Moreover, any such effect on a subset of the cohort may introduce bias in the LME model, thus also affecting other patients.

First, we checked for a systematic difference in the SBS5 mutation rate in patients with and without SBS-MM1. No such difference could be found.

```
# comparing mutation rate per year in melphalan-treated and untreated patients, as an initial check for bias due to SBS-MM1

# samples with MM1 identified
samples_mm1 <- names(sample.sigt.profs[sapply(sample.sigt.profs, function(x) ifelse("SBS.MM1" %in% x, TRUE, FALSE))])

# fit LME model for all patients
muts.per.year.lmer <- lmer(Num.mutations ~ 0 + Age + (Age + 0 | Sample), data=allsamples.df, REML=FALSE)

# calculate mutation rate
mutrate_patients <- distinct(clin, sample_ID, Age)
mutrate_patients$Est.mut.rate.per.Gb.per.year <- (fixef(muts.per.year.lmer)["Age"] + 
                                                    ranef(muts.per.year.lmer)$Sample[mutrate_patients$sample_ID,"Age"]) / 6

mutrate_patients <- mutrate_patients %>%
    mutate(SBS_MM1 = ifelse(sample_ID %in% samples_mm1, "Present", "Absent")) %>%
    filter(!is.na(Est.mut.rate.per.Gb.per.year))

# test for difference in mutation rate
wilcox_p <- wilcox.test(filter(mutrate_patients, SBS_MM1 == "Present")$Est.mut.rate.per.Gb.per.year,
                        filter(mutrate_patients, SBS_MM1 == "Absent")$Est.mut.rate.per.Gb.per.year)$p.value

# Graphical data presentation
ggplot(mutrate_patients, aes(SBS_MM1, Est.mut.rate.per.Gb.per.year))+
  geom_jitter()+
  geom_boxplot(fill = NA, outlier.shape = NA)+
  labs(x = "SBS-MM1 status",
       y = "Estimated SBS5 mutation rate\nmutations/Gb/year")+
  annotate("text", x = 1.5, y = 7.8, size = 6, col = "darkred",
           label = paste0("p = ", round(wilcox_p, 3)))+
  theme(text = element_text(size = 15))
```

Second, we fit a new LME model to estimate mutation rate in patients without SBS-MM1, comparing the estimated time of the first multi-gain event with estimates from the main LME model trained on all patients. The results vere virtually identical, showing that patients with SBS-MM1 do not affect the overall estimation of mutation rate using LME models.

```
# samples with MM1 identified
samples_mm1 <- names(sample.sigt.profs[sapply(sample.sigt.profs, function(x) ifelse("SBS.MM1" %in% x, TRUE, FALSE))])

# samples without MM1
data_nomm1 <- filter(allsamples.df, !Paz %in% samples_mm1)

# fit LME model for all patients
muts.per.year.lmer <- lmer(Num.mutations ~ 0 + Age + (Age + 0 | Sample), data=allsamples.df, REML=FALSE)
muts.per.year.lmer.nomm1 <- lmer(Num.mutations ~ 0 + Age + (Age + 0 | Sample), data=data_nomm1, REML=FALSE)


multigains.nomm1 <-  estimateGains(clin = clin, 
                                   time_windows_mol_time = time_windows_mol_time, 
                                   allsamples.mrca=allsamples.mrca,
                                   muts.per.year.lmer = muts.per.year.lmer.nomm1)

multigains.all <-  estimateGains(clin = clin, 
                                 time_windows_mol_time = time_windows_mol_time, 
                                 allsamples.mrca=allsamples.mrca,
                                 muts.per.year.lmer = muts.per.year.lmer)
```

```
nomm1_firstgain <- multigains.nomm1 %>%
  filter(variable == 'estimate', 
         time_window == "first") %>%
         dplyr::select(Sample, Est.mut.rate.per.Gb.per.year, age_gain) %>%
         dplyr::rename(nomm1_mutrate = "Est.mut.rate.per.Gb.per.year",
                       nomm1_agegain = "age_gain")

firstgain <- multigains.all %>%
  filter(variable == 'estimate', 
         time_window == "first",
         Sample %in% nomm1_firstgain$Sample) %>%
         dplyr::select(Sample, Est.mut.rate.per.Gb.per.year, age_gain) %>%
         dplyr::rename(full_mutrate = "Est.mut.rate.per.Gb.per.year",
                       full_agegain = "age_gain")

compare_rate <- left_join(nomm1_firstgain,
                          firstgain)
```

```
## Joining, by = "Sample"
```

```
rate_plot <- ggplot(compare_rate, aes(x = full_mutrate, y = nomm1_mutrate))+
    geom_segment(x = 0, y = 0, xend = 10, yend = 10, size = 1, col = "darkgray")+
    scale_x_continuous(limits = c(0,10))+
    scale_y_continuous(limits = c(0,10))+
    geom_point(size = 2)+
    labs(x = "Mutations/Gb/year\n(Full model)",
         y = "Mutations/Gb/year\n(No SBS-MM1)")+
    theme(text = element_text(size=15)) 

age_plot <- ggplot(compare_rate, aes(x = full_agegain, y = nomm1_agegain))+
    geom_segment(x = 0, y = 0, xend = 50, yend = 50, size = 1, col = "darkgray")+
    scale_x_continuous(limits = c(0,50))+
    scale_y_continuous(limits = c(0,50))+
    geom_point(size = 2)+
    labs(x = "Age at the first multi-gain event\n(Full model)",
         y = "Age at the first multi-gain event\n(No SBS-MM1)")+
    theme(text = element_text(size=15)) 

ggarrange(rate_plot, age_plot, nrow = 1)
```

Taken together, there was no evidence that bleeding of mutations between SBS-MM1 and SBS5 affected the estimated timing of events.
