# Supplementary material for: Timing the initiation of multiple myeloma
Source: Nat Commun. 2020 Apr 21;11:1917. doi: 10.1038/s41467-020-15740-9 (PMC7174344; doi:10.1038/s41467-020-15740-9)
Supplement: Supplementary file 13 — Reporting summary [file 41467_2020_15740_MOESM13_ESM.pdf]

nature research

Corresponding author(s): Francesco Maura

Last updated by author(s): 2020/03/09

Reporting Summary

Nature Research wishes to improve the reproducibility of the work that we publish. This form provides structure for consistency and transparency in reporting. For further information on Nature Research policies, see Authors & Referees and the Editorial Policy Checklist .

Statistics

For all statistical analyses, confirm that the following items are present in the figure legend, table legend, main text, or Methods section.

n/a	Confirmed	
☐	☒	The exact sample size (*n*) for each experimental group/condition, given as a discrete number and unit of measurement
☐	☒	A statement on whether measurements were taken from distinct samples or whether the same sample was measured repeatedly
☐	☒	The statistical test(s) used AND whether they are one- or two-sided<br>*Only common tests should be described solely by name; describe more complex techniques in the Methods section.*
☐	☒	A description of all covariates tested
☐	☒	A description of any assumptions or corrections, such as tests of normality and adjustment for multiple comparisons
☐	☒	A full description of the statistical parameters including central tendency (e.g. means) or other basic estimates (e.g. regression coefficient) AND variation (e.g. standard deviation) or associated estimates of uncertainty (e.g. confidence intervals)
☐	☒	For null hypothesis testing, the test statistic (e.g. *F*, *t*, *r*) with confidence intervals, effect sizes, degrees of freedom and *P* value noted<br>*Give P values as exact values whenever suitable.*
☐	☒	For Bayesian analysis, information on the choice of priors and Markov chain Monte Carlo settings
☒	☐	For hierarchical and complex designs, identification of the appropriate level for tests and full reporting of outcomes
☒	☐	Estimates of effect sizes (e.g. Cohen's *d*, Pearson's *r*), indicating how they were calculated

Our web collection on statistics for biologists contains articles on many of the points above.

Software and code

Policy information about availability of computer code

Data collection	Publicly available published software were used for whole genome sequencing analysis as follows BWA-MEM v0.7.8, Caveman v1.13.2, MuSE v1.0, Strelka v1.0.13, Seurat v2.6, MuTect v2.2, Pindel v1.5.7, BRASS v4.012, Ascat v1.5.2, Battenberg
Data analysis	Pylogenetic tree recontruction: hierarchical Dirichlet (https://github.com/Wedge-Oxford/dpclust) process and PhyloWGS (https://github.com/morrislab/phylowgs);<br>molecular time code (https://github.com/nicos-angelopoulos/mol_time);<br>mutational signatures were analyzed by SigProfiler (https://github.com/AlexandrovLab/SigProfilerExtractor), hierarchical Dirichlet process (https://github.com/nicolaroberts/hdp) and a new code released with the paper (https://github.com/evenrus/mmsig)

For manuscripts utilizing custom algorithms or software that are central to the research but not yet described in published literature, software must be made available to editors/reviewers. We strongly encourage code deposition in a community repository (e.g. GitHub). See the Nature Research guidelines for submitting code & software for further information.

Data

Policy information about availability of data

All manuscripts must include a data availability statement. This statement should provide the following information, where applicable:

- Accession codes, unique identifiers, or web links for publicly available datasets
- A list of figures that have associated raw data
- A description of any restrictions on data availability

All data are available in the following public depositories: phs000748.v1.p1, phs000348.v2.p1 , EGAS00001001692, EGAD00001001898, EGAS00001003709, GSE117156

Field-specific reporting

Please select the one below that is the best fit for your research. If you are not sure, read the appropriate sections before making your selection.

- [x] Life sciences
- [ ] Behavioural & social sciences
- [ ] Ecological, evolutionary & environmental sciences

For a reference copy of the document with all sections, see nature.com/documents/nr-reporting-summary-flat.pdf

Life sciences study design

All studies must disclose on these points even when the disclosure is negative.

Sample size	In this study we included 89 and 973 multiple myeloma patients with available genome and exomes sequencing data respectively. Additional 107 and 89 B-cell lymphomas and chronic lymphocytic leukemia were included. Sample size was defined according to the sequencing data availability.
Data exclusions	Only samples turning out to have tumor cell content below the limited described in the paper were excluded from the series. For the molecular clock analysis patients with mutational burden >10.000 SNVs and/or ploidy >4 were excluded.
Replication	All data can be entirely reproduced. All the sequencing data and bio-informatics codes are available in public depository
Randomization	The study was not an experimental clinical treatment trial and hence no randomization was performed.
Blinding	NA

Reporting for specific materials, systems and methods

We require information from authors about some types of materials, experimental systems and methods used in many studies. Here, indicate whether each material, system or method listed is relevant to your study. If you are not sure if a list item applies to your research, read the appropriate section before selecting a response.

Materials & experimental systems

n/a	Involved in the study	
x		Antibodies
x		Eukaryotic cell lines
x		Palaeontology
x		Animals and other organisms
	x	Human research participants
	x	Clinical data

Methods

n/a	Involved in the study	
x		ChIP-seq
x		Flow cytometry
x		MRI-based neuroimaging

Human research participants

Policy information about studies involving human research participants

Population characteristics	The study involved the use of human samples, which were collected after written informed consent was obtained (WTSI protocol number 15/046). Samples and data were obtained and managed in accordance with the Declaration of Helsinki. DNA was extracted from CD138+ cells purified from the bone marrows of 30 patients. Twenty-six (86%) patients had more than one sample collected at different time points for a total of 67 tumor samples and 30 matched normals collected from peripheral blood mononuclear cells. This cohort was previusly published and characterized in another paper (https://doi.org/10.1038/s41467-019-11680-1).<br>To this cohort we added available whole genome sequencing data and two existing and published cohort of whole exome sequencing.
Recruitment	we collected available genomic data from public depository. The enrollment criteria were: patients with multiple myeloma and available genomic (WGS or WXS) data.
Ethics oversight	Memorial Sloan Kettering IRB 14-276 and Wellcome sanger Institute 15/046

Note that full information on the approval of the study protocol must also be provided in the manuscript.

Clinical data

Policy information about clinical studies

All manuscripts should comply with the ICMJEguidelines for publication of clinical research and a completedCONSORT checklist must be included with all submissions.

Clinical trial registration	NCT01454297
Study protocol	https://clinicaltrials.gov/ct2/show/NCT01454297
Data collection	Multi-centers prospective collection of Bone marrow and Peripheral Blood from newly diagnosed and relapsed multiple myeloma patients
Outcomes	The primary objective of this observational study is to identify the molecular profiles and clinical characteristics that define subsets of myeloma patients during the course of the disease.
